# The Italian epistemic marker *mi sa* [to me it knows] compared to *so* [I know], *non so* [I don’t know], *non so se* [I don’t know whether], *credo* [I believe], *penso* [I think]

**DOI:** 10.1371/journal.pone.0274694

**Published:** 2022-09-22

**Authors:** Ilaria Riccioni, Andrzej Zuczkowski, Roberto Burro, Ramona Bongelli

**Affiliations:** 1 Department of Education, Cultural Heritage and Tourism, University of Macerata, Macerata, Italy; 2 Department of Human Sciences, University of Verona, Verona, Italy; 3 Department of Political Science, Communication and International Relations, University of Macerata, Macerata, Italy; Potsdam University, GERMANY

## Abstract

The two studies presented in this paper concern the Italian epistemic marker *mi sa* [lit. to me it knows], which seems to have no equivalent in other European languages and has received very little attention in the literature. No analysis of the occurrences of *mi sa* in contemporary spoken corpora can be found (first gap) as well as no investigation on the epistemic relationship between *mi sa* and (1) the other modal expressions that use the verb *sapere* [to know] in the first person singular of the simple present, i.e., *so* [I know], *non so* [I do not know], *non so se* [I do not know whether] as well as (2) its supposed synonyms *credo* [I believe] and *penso* [I think] (second gap). The two studies are closely intertwined, the first being an exploratory, qualitative pilot study for the second. Study 1 aims to fill the first gap through the analysis of the contemporary Italian spoken corpus *KIParla*. The quantitative and qualitative analyses revealed five types of occurrences (theoretically reducible to two main ones), the most numerous of which are ‘*mi sa che* + proposition’. Study 2 aims to fill the second gap through a questionnaire administered online. The quantitative and statistical results showed the epistemic relationships between the six markers: for the majority of the participants, in the epistemic continuum that goes from unknowledge to uncertainty and then to knowledge, (1) *non so* refers to unknowledge; *non so se*, *mi sa*, *credo* and *penso* refer to uncertainty; *so* refers to knowledge; (2) *mi sa*, *credo*, *penso* confirm to be synonyms; (3) *non so se* is evaluated as much more uncertain than *mi sa*, *credo*, *penso*. These four epistemic markers seem to occupy a different position along the uncertainty continuum ranging between two poles: doubt (high uncertainty) and belief (low uncertainty).

## Introduction

For many years a wide range of studies, from different disciplines related to written and spoken language, have focused on the *epistemic stance*, that has been differently defined by researchers. For some of them, the process of epistemic stance taking is closely related to the speaker/writer’s commitment toward the truth value of the piece of information conveyed (*epistemicity*; e.g. [1–4]), for some others, also to the speaker/writer’s orientation towards the source of knowledge (*evidentiality*; e.g. [5–9]).

One of the most widely accepted definitions of epistemic stance is that of Ochs [5], according to which it “refers to knowledge or belief vis-à-vis some focus of concern, including degrees of Certainty or knowledge, degrees of commitment to truth of propositions, and sources of knowledge, among other epistemic qualities”. This definition is consistent with ours, according to which “the epistemic stance taking concerns the positions both epistemic (commitment) and evidential (source of information) which speakers/writers take during communication in regards to the information they are conveying and which they express through lexical and morphosyntactic means” [8] (p. 3).

As for the Italian language, many scholars are interested in the study of singular aspects of epistemicity and evidentiality, such as epistemic future (e.g. [10–15]); epistemic adverbs (e.g. [16–21]); mental verbs (e.g. [22, 23]); modal verbs (e.g. [24–29]); modality in a broad sense (e.g. [30–32]) and lexical vs grammatical aspects (e.g. [33]). Although, therefore, a lot of work has been carried out on the Italian epistemic and evidential markers, it can be noted that, among them, *mi sa* has been the subject of very few investigations.

*Sa* [(he/she/it) knows], in Italian grammar, is the third person singular in the present indicative of the verb *sapere*, which as a full lexical verb can have a double meaning, either cognitive or gustative: *sapere* [to know] (i.e., *lui sa sempre tutto* [he always knows everything]) and *aver sapore* [to have taste, to taste] (i.e., *questo vino sa di aceto* [this wine tastes like vinegar]).

The impersonal verb expression *mi sa* [literally, ‘to me it knows’], often followed by a completive proposition introduced by *che* [that], is an epistemic marker which communicate the speaker’s *personal belief*, *opinion*, *impression*, as *credo* [I believe], *penso* [I think], *mi pare* [it appears to me], *mi sembra* [it seems to me], *ho l’impressione* [I have the impression]. *Mi sa* can be usually found also in responses to yes/no questions, in the expression *Mi sa di sì/no* [literally, ‘To me it knows of yes/no’, meaning ‘I think so/I do not think so’].

The bound with the gustative meaning of the verb *sapere* [to have taste, to taste], in a metaphorical use, can be found in the expression *mi sa di* [literally: to me it tastes of, which can be translated with ‘it sounds/looks/smells to me’] plus an adjective, i.e., *mi sa di vecchio* [it smells of old to me] or a noun, i.e., *mi sa di truffa* [it sounds of a trick to me].

In this article, for *mi sa* we will sometimes use the literal translation ‘to me it knows’, which is ungrammatical in English, instead of the grammatically acceptable translation ‘to me it is known’, since this latter is a marker of knowledge/certainty both in English and in Italian (*a me è noto*), while *mi sa* is a marker of believing/uncertainty. Nevertheless, when in Study 1, section Qualitative results, we translate into English the different Italian examples of *mi sa* taken from the spoken corpus, in order to make such excerpts more readable, we will try to adapt the translation of *mi sa* to the context, using synonymic expressions (I think, I believe, I guess, I feel like, it seems to me, it sounds to me, to me it looks like, etc.)

Going back to the epistemic meaning of *mi sa*, in the following example,

(1)

X:  *Dov’è Andrea*?

  Where is Andrea?

Y:  ***Mi sa*** *che sta andando a Verona*

  **To me it knows** (= I believe/think, etc.) that he is going to Verona

Y is epistemically saying that they *are not certain* that, i.e., they do not know whether, the piece of information included in the completive proposition p (= Andrea is going to Verona) is *true*, but they *believe* it is. In example (1), *mi sa* could be replaced by *credo* [I believe] or *penso* [I think] indifferently.

*Mi sa* is therefore a modal communicating the speaker’s epistemic position of *uncertainty*, *believing*, even though it includes the verb *sapere* [to know], which usually functions as a modal referring to *knowledge*, *certainty*. Suppose that in the above example (1) Y answers X’s question by saying

Y:  *So che sta andando a Verona*

   I know that he is going to Verona

In this case, Y is epistemically saying that the piece of information p (= Andrea is going to Verona) is *certain*, *known* to them, which implies that p is also *true* for them.

As far as we know, the expression *mi sa che* + proposition seems to have no equivalent in other European languages such as English, French, Spanish, German, Romanian, Polish, Flemish etc. (see the Acknowledgments). *Mi sa* seems therefore to be a linguistic phenomenon pertaining only to the Italian language, typical of it: also for this reason, we find the deepening of our knowledge about this epistemic marker particularly interesting. *Mi sa*, as an epistemic marker, has received very little attention in the literature. Some exceptions are Bozzone Costa [34], Orletti [35], D’Achille [36], and Cialdini [37].

The most systematic work, even though brief, is by Serianni [38] who considers *mi sa* as the most immediate and spontaneous way for speakers to express their *opinion* in contemporary spoken Italian [38] (p. 18), therefore as a simple synonym of *credo* [I believe] or *penso* [I think] [38] (p. 19). The main differences between *mi sa* and *credo*/*penso* are that the former has a more informal, colloquial, familiar use [38] (p. 19) and even more morphological restrictions: as a matter of fact its use is limited to

1. the speaker’s I (*mi* [to me]), i.e., the first person singular only: the other persons (second and third, singular and plural) are ungrammatical: *ti/vi sa [to you it knows]; *gli/le/a loro/a Paolo sa [to him/her/them/Paul it knows] and
2. the simple present tense (*sa* [(it) knows]): any other tense is ungrammatical: past *mi sapeva [to me it knew], future *mi saprà [to me it will know], etc.;
3. even the verb of the completive proposition introduced by *mi sa* is normally in the indicative mood, seldom in the conjunctive [38] (p. 19).

On the contrary, *credo* [I believe], *penso* [I think] and the other *verba opinandi* such as *suppongo* [I suppose], *ipotizzo* [I assume], etc. can be used with any person and tense, and the verb of the proposition that they introduce is normally in the conjunctive mood, not in the indicative.

Serianni’s analysis of the occurrences of *mi sa* is limited to two written corpora, literary and journalistic: he quotes 42 examples of occurrences of *mi sa* taken from the *Primo Tesoro della Lingua Letteraria Italiana del Novecento in DVD* [39] and from the newspaper “Il Corriere della Sera”, year 2011, available online (https://archivio.corriere.it/Archivio/interface/landing.html). No analysis of the occurrences of *mi sa* in contemporary spoken corpora can be found, neither in Serianni’s paper [38] nor in the literature, as well as no study has investigated the epistemic relationships between *mi sa* and the other modal expressions that use the verb *sapere* [to know] in the first person singular of the simple present, i.e., *so* [I know], *non so* [I do not know], *non so se* [I do not know whether].

Finally, Serianni [38] states that *mi sa*, *credo* [I believe] and *penso* [I think] are synonyms. This judgment is based on his own intuitive linguistic competence as an Italian native speaker and eminent linguist, but is that enough? What kind of *evidence* can be given to support Serianni’s judgment?

This article aims to answer these questions and investigate the characteristics and distinctive traits of the epistemic marker *mi sa* through two interconnected studies.

Study 1 seeks to investigate occurrences and concrete linguistic uses of *mi sa* through a mainly qualitative analysis conducted on a corpus of contemporary spoken Italian (KIParla [40]).

Study 2 intends to analyse the relationships between *mi sa* and other epistemic markers of the Italian language such as *so* [I know], *non so* [I do not know], *non so se* [I do not know whether], *credo* [I believe] and *penso* [I think], through a questionnaire administered to a sample of Italian speakers. Some results of the analyses conducted in Study 1 informed the construction of the questionnaire stimuli.

### Theoretical framework

The theoretical framework of our two studies is a model of epistemic stance [8, 41, 42] according to which speakers can communicate each single piece of information either as *known/certain* or *uncertain* or *unknown* to them, i.e., they can communicate a propositional content from one of the following three epistemic positions: *knowing/certain*, *uncertain* and *unknowing*.

Each of the six modal markers under analysis in Study 2 refers to one of such positions. In order to suppose which are the epistemic relationships among the six markers, in the following their epistemic positions will be specified.

In the Introduction, example (1) showed that, when the speaker Y says *so che p* [I know that p], where p is ‘Andrea is going to Verona’, they presuppose that p is true, while when they say *mi sa che p* [to me it knows that p], they communicate not to know whether p is true or false but *to believe* that p is true, i.e., they communicate that the probability that p is true is higher than the probability that p is false. Therefore, *so* [I know] is a marker of *knowledge/certainty*, while *mi sa* [to me it knows] is a marker of *uncertainty/believing*, similar to *credo* [I believe] and *penso* [I think]:

***so che p* [I know that p]** → **speaker’s knowing/certain position**

***mi sa*, *credo*, *penso che p* [to me it knows, I believe, I think that p]** → **speaker’s uncertain position**

Let’s now take *non so* [I do not know] into consideration.

This expression, as an “epistemic disclaimer” can have two different epistemic meanings, depending on its place in a conversational sequence; it can communicate either “no-knowledge” or “insufficient knowledge” [43–50].

Following our theoretical model, these two different epistemic meanings of *non so* [I don’t know] depend also on whether *non so* is followed by the conjunction *se* [if/whether] (e.g., *Non so se Andrea sta andando a Verona* [I do not know whether Andrea is going to Verona] which was used in the questionnaire in Study 2) or by an interrogative adverb (*dove* [where], *come* [how], *quando* [when], *perché* [why]) or by an interrogative pronoun or adjective (*chi* [who], *che cosa* [what], *quale* [which]), i.e., by one of those words that in English are called wh-words (e.g., *Non so perché Andrea sta andando a Verona* [I do not know why Andrea is going to Verona] which was used in the questionnaire in Study 2).

Both the examples *non so se p* [I do not know whether p] and *non so perché p* [I do not know why p] in some conversational sequences can function as indirect interrogative sentences, corresponding to a polar question (*Andrea sta andando a Verona*? [Is Andrea going to Verona?]) and a wh-question (*Perché Andrea sta andando a Verona*? [Why is Andrea going to Verona?]), respectively. The declarative sentence *Non so perché Andrea sta andando a Verona* [I do not know why Andrea is going to Verona] presupposes that the proposition p *Andrea is going to Verona* is known to the speaker and thus true and it communicates that the speaker does not know the reasons or purposes of Andrea’s going there. Therefore, the sentence *Non so perché p* [I do not know why p] communicates a speaker’s *lack of knowledge*, i.e., an *unknowledge*, about the meaning of the only element (*perché* [why]) which is unknown to them within a state of affairs presupposed as true, i.e., as known and certain to them. The word *perché* [why] is a pro-form for the missing information. That is the reason why, in our epistemic model, the sentence *Non so perché Andrea sta andando a Verona* [I do not know why Andrea is going to Verona], as well as the corresponding direct question *Perché Andrea sta andando a Verona*? [Why is Andrea going to Verona?] communicate the speaker’s *unknowing* position:

***non so perché p* [I do not know why p]** → **speaker’s unknowing position**

On the contrary, the sentence *Non so se Andrea sta andando a Verona* [I do not know whether Andrea is going to Verona], more than a lack of knowledge expresses a *lack of certainty* (= uncertainty) about the *truth* of the *whole* proposition p: the speaker is saying that they do not know whether p is true or false. Differently from *Non so perché Andrea sta andando a Verona* [I do not know why Andrea is going to Verona], the sentence *Non so se Andrea sta andando a Verona* [I do not know whether Andrea is going to Verona] does not presuppose that p is true but conveys that p is *possibly* true [51]. If p is possibly true, it is also possibly false. Thus, the sentence leaves two possibilities open, i.e., two alternatives, that p is true or that p is false. If p is true, it means that Andrea is going to Verona. If p is false, it means that Andrea is *not* going to Verona, i.e. that *non p* is true. Since non p is the negative opposite of p, the alternatives in the sentence *Non so se Andrea sta andando a Verona*, besides being thought of as *p is true or p is false*, may also be thought of as *p or non p*, as in the sentence *Non so se Andrea sta andando a Verona* ***o no*** [I do not know whether Andrea is going to Verona **or not]**. The difference between the two sentences is that in *Non so se Andrea sta andando a Verona* the negative alternative *non p* remains implicit [***non so se p (o non p)***], while in the sentence *Non so se Andrea sta andando a Verona* ***o no*** [I do not know whether Andrea is going to Verona **or not]** the negative alternative *non p* is explicit, lexicalized as *o no* [or not]. Uncertainty, by definition, implies *alternatives*: the speaker is faced with two different possibilities (p and non p), the first being explicit, lexicalized (Andrea is going to Verona), the second being implicit, not lexicalized (Andrea is not going to Verona).

In our reading of the sentence *Non so se Andrea sta andando a Verona* [I do not know whether Andrea is going to Verona], both alternatives are communicated as having the same probability to be true. It is thus a matter of *doubt* for the speaker, in the sense that they communicate *to be in doubt* between p and non p, i.e., between the truth or the falseness of p. That is the reason why, in our epistemic model, the sentence *Non so se Andrea sta andando a Verona* [I do not know whether Andrea is going to Verona], as well as the corresponding direct question *Andrea sta andando a Verona*? [Is Andrea going to Verona?], communicate the speaker’s *uncertain* position:

***non so se p* [I do not know whether p]** → **speaker’s uncertain position**

**Fig 1:**
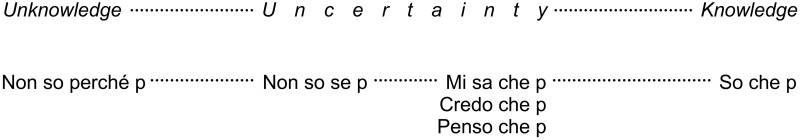
The six sentences along the epistemic continuum.

The epistemic continuum corresponding to the six sentences could be represented *horizontally* in the following way:

In Fig 1, unknowledge is represented as contiguous to uncertainty as well as uncertainty to knowledge, i.e., uncertainty is in between unknowledge and knowledge.

Let us comment first on the left side of Fig 1: that unknowledge is contiguous to uncertainty means that the sentence *Non so perché p* [I do not know why p] and the sentence *Non so se p* [I do not know whether p] are adjacent. Both sentences include the expression *Non so* [I do not know] and in this strict sense they both include unknowledge. But the difference between the two sentences is made by what follows the expression *Non so* [I do not know], i.e., by *perché* [why] and *se* [whether]. In *Non so perché p* [I do not know why p] the speaker’s unknowledge refers to *one single element* (*perché* [why]), while in *Non so se p* [I do not know whether p] the speaker’s unknowledge refers to the truth or falseness of *two* alternatives, *two* propositions, and under these conditions unknowledge becomes uncertainty, the former changes into the latter. Roughly speaking, unknowledge communicated through language is *single*, uncertainty is *double*.

Moreover, in the middle of Fig 1, uncertainty is represented as ranging between two poles, from high uncertainty (the *not knowing whether* pole) to low uncertainty (the *believing* pole). The sentence *Non so se p* [I do not know whether p] refers to the pole of high uncertainty and is supposed to be *more uncertain* than the sentences *Mi sa*/ *Credo*/ *Penso che p* [To me it knows/ I believe/ I think that p], which refer to the pole of low uncertainty, since in our reading the former sentence communicates a *doubt* of the speaker (which can be paraphrased as *I do not know whether p is true or false*, i.e., p is communicated as having the same probability of being true as of being false), while the three latter sentences deliver not a doubt but an opinion, a supposition, an assumption, a belief and the like (which can be paraphrased as *I do not know whether p is true or false*, *but I am inclined to believe that p is true*, i.e., p is communicated as having more probability of being true than false).

Finally, the right side of Fig 1 shows the contiguity of the marker of uncertainty/believing *mi sa* to the marker of knowledge/certainty *so* [I know].

### Aims

The present paper intends to deepen Serianni’s work [38] in a twofold direction, the first concerns aim (1), the second aims (2)-(4):

1. **Occurrences of *mi sa* in a contemporary Italian spoken corpus**
  The first aim is to extend the analysis of the occurrences of *mi sa* to an Italian contemporary spoken corpus; thus the first research question is: how many are such occurrences in such corpus? And of what types are they, i.e., which are their syntactic and semantic features?
  Study 1 was conceived to answer this question.
2. ***Mi sa* compared to *so*, *non so*, *non so se***
  The second research question is: which is the epistemic relationship between *mi sa* and the other modal expressions that use the verb *sapere* [to know] in the first person singular of the simple present, i.e., *so* [I know], *non so* [I do not know], *non so se* [I do not know whether]? In particular, which epistemic positions of the speaker do such markers communicate?
3. ***Mi sa* compared to *credo* and *penso***
  The third research question is: which is the epistemic relationship between *mi sa*, *credo* [I believe] and *penso* [I think], in particular what kind of evidence can be given to support Serianni’s (2012) judgement that the three expressions are synonyms?
4. ***Mi sa*, *credo*, *penso* compared to *non so se***
  Within the specific epistemic continuum of uncertainty (see Fig 1), which are the epistemic relationships between *non so se* [I do not know whether], on the one side, and *mi sa*, *credo* and *penso*, on the other? Is the degree of uncertainty that they communicate the same or is it significantly different?
  Study 2 was conceived to answer these three questions.

## Study 1. Occurrences of *mi sa* in contemporary spoken corpora

### Methods and materials

#### Corpus

To address research question (1), the distribution of *mi sa* was investigated by querying the contemporary Italian spoken corpus *KIParla* [40], which is an upgradable corpus collecting more than 100 hours of conversations in Italian, freely accessible at the following link: https://kiparla.it. KIParla is composed of two modules: *KIP* [52] and *ParlaTO* [53].

#### Procedures

In order to identify the distribution of *mi sa*, the two corpus modules (KIP and ParlaTO) were queried separately, using the simple search strings “*mi sa”* (last accessed 29th of April 2021).

### Results

#### Quantitative results

The queries returned 74 matches of *mi sa* in KIP and 21 in ParlaTO, which were then verified manually in order to check that all were indeed epistemic expressions. 1 occurrence was eliminated (*mi sa dire qualcosa sull’institore*? [can you tell me something about the general manager of the company?]), since in this case *mi sa [dire]* does not mean *to me it knows/I think*, but it refers to the second person of the interlocutor (*lei* [you]) and means ‘can you/are you able [to tell me]’. Therefore, the total occurrences are 94 (74 in KIP and 20 in ParlaTO, see Table 1).

**Table 1:** Occurrences of *mi sa* in KIP and ParlaTO.

Mi sa						
	KIP		ParlaTO		Tot.	
	freq	%	freq	%	freq	%
Mi sa che + prop.	46	62.2	11	55	57	60.6
Mi sa parenthetical	20*	27	5**	25	25	26.6
Mi sa + elliptical prop.	4	5.4	1	5	5	5.3
Mi sa che… (pending)	2	2.7	2	10	4	4.3
Mi sa di (metaphorical)	2	2.7	0	0	2	2.1
Mi sa di no	0	0	1	5	1	1.1
**Tot**.	74	100	20	100	94	100

* 11 medial position, 9 final position

** 1 medial position, 4 final position

**Fig 2:**
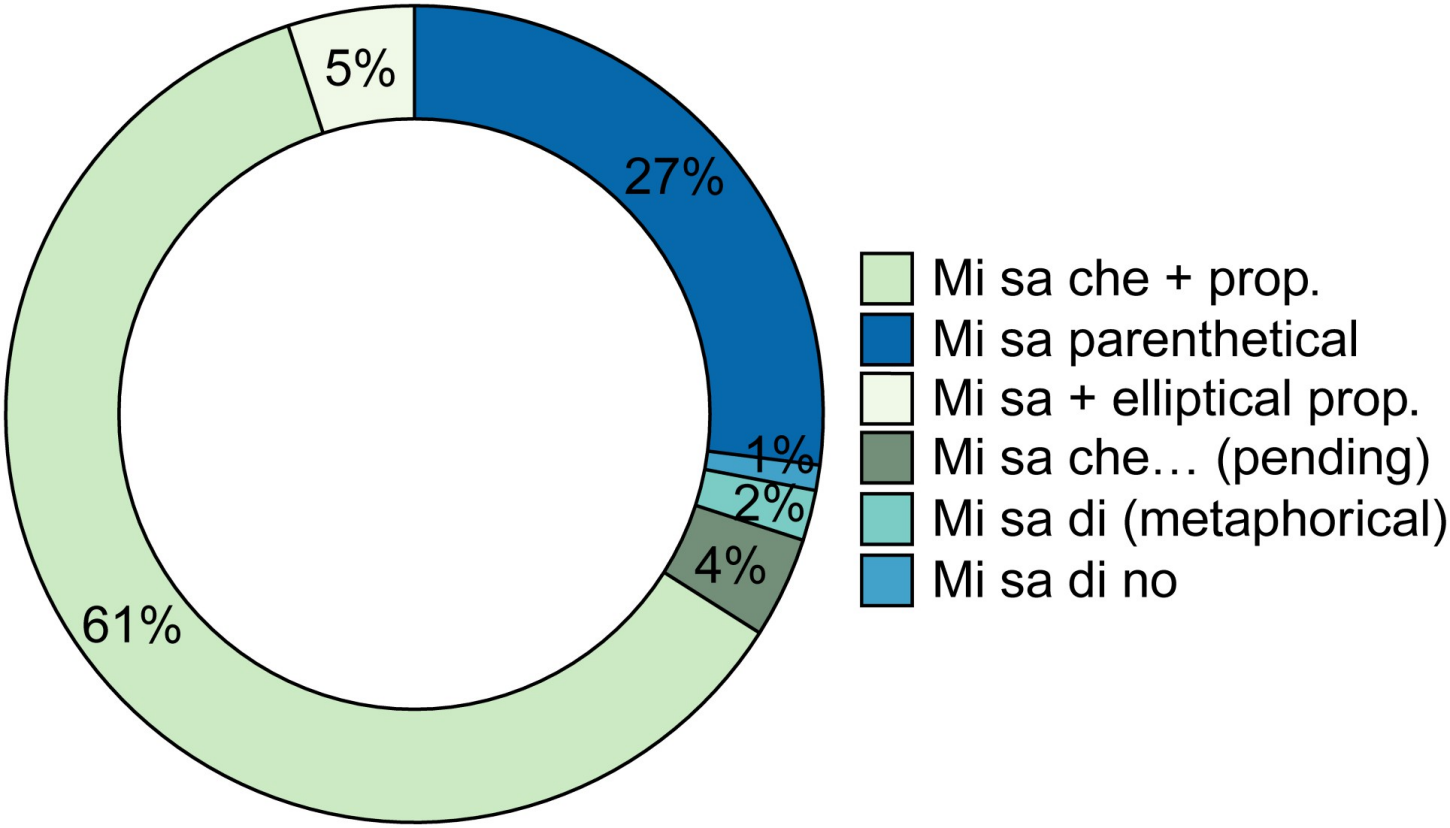
Percentages of the occurrences of *mi sa* in both modules (KIP + ParlaTO).

In his analysis of the written corpora, Serianni [38] focused mostly on *mi sa che* + proposition. The analysis of the spoken corpus revealed six types of structures, the most numerous of which are *mi sa che* + proposition (60.6%) and *mi sa* parenthetical (26.6%), as shown in Table 1 and Fig 2:

*Mi sa che* [(lit. to me it knows that) I think/guess…that] followed by a proposition;

*Mi sa* parenthetical, both in medial and final position;

*Mi sa* followed by an ‘elliptical’ proposition;

*Mi sa che…* left ‘pending’ by the speaker;

*Mi sa di* [(lit. to me it tastes of) he/she/it looks/sounds/smells like (…) to me] followed by an adjective or a noun (in a metaphorical use);

*Mi sa di no* [(lit. to me it knows of no) I do not think so/I guess not].

#### Qualitative results

Here are some examples of each type of the identified structures, presented within the conversational sequences in which they are inserted. For each example we provide a brief, mainly descriptive, analysis in which we attempt to account for the epistemic function of the marker *mi sa*, also in relation to the sequential context. The English translation is a representation as faithful as possible of the original colloquial Italian and, as anticipated in the Introduction, *mi sa* has been translated by trying to adapt its meaning to the context, using from time to time synonymic expressions as I think, I believe, I guess, I feel like, it seems to me, it sounds to me, to me it looks like, etc. For the transcription, in the corpus a simplified version of the Jefferson system [54] has been used.

*Mi sa che + proposition*. The following example is extracted from a natural, informal conversation between two female participants (both of them under 25).

(2)

[KIP, BOA3001]

BO002:  *che ore sono*?

      what time is it?

BO003:  *non lo so*

      I don’t know

BO002:  *perché* ***mi sa che*** *(*.*) mi si è fermato l’orolo*:*gio*

      because I think that my watch stopped

The expression *mi sa che* is a part of the utterance in which BO002 explains the reason why, in her previous turn, she asked “what time is it?”: she believes, suspects, has the impression that her watch has stopped. In this short sequence, following the theoretical model previously described, we can at first observe the displaying of an unknowing position by both the participants, respectively conveyed by BO002’s wh-question and BO003’s epistemic disclaimer “I don’t know”. In the third turn BO002, giving reasons for her previous question, shift into an uncertain, believing position.

*Mi sa che… (pending)*. Excerpt (3) is taken from a semi-structured interview, with four participants (age range: 26–30), one female interviewer, two male interviewees and another male participant (TOR002, who has only a few speaking turns and seems to have a supporting role for the interviewer); the main topic of the interview is work.

(3)

[ParlaTO, PTD021]

TOI012:  *e quando ho firmato il contratto a tempo indeterminato lacrimoni e[*::*]*

      and when I signed the permanent contract tears an[d

TOR001:          *[certo ci credo]*

             [sure I believe that]

TOR002:          *[e*::*]*:*h*

             [e]h

TOI012:   *festa*

       celebration

TOR001:  *okay*,

     okay,

TOR001:  *la domanda dopo è lavori in proprio o sei dipendente ma[*: ***mi sa che****]*

     then the question is are you self-employed or are you employed but [I believe that

TOI012:          *[beh dipenden]te*,

               [well emplo]yed,

TOI013:          *[dipendente*. *cer(to)]*

               [employed. su(re)]

The interviewer TOR001 is asking about the interviewees’ type of job (self-employment/employment) and they both respond simultaneously, overlapping in a transition relevance place. *Mi sa che* comes at the end of a sentence left unsaid and it follows the adversative conjunction *ma* [but]. We can assume that, in this case, *mi sa che* remains pending, not only because of the overlapping speech of the two participants, but the presence of ‘but’, together with the content of the preceding turns, suggest that TOR001 probably came to the conclusion on her own. In fact, TOI012 has just said that he signed a permanent contract, typical of employment (not self-employment). TOR001, in the same turn, shifts from a Not Knowing Whether (expressed by the alternative question) to a Believing position (conveyed by *mi sa che*), with a self-correction, where what is believed (e.g. “the answer to this question is already implicit in what you said before”) is left unsaid.

*Mi sa + elliptical proposition*. Compared to the type of occurrences that in Table 1 we named as *mi sa che* + proposition, the type of occurrences named as *mi sa + elliptical proposition* refers to those occurrences in which, after *mi sa* both the conjunction *che* [that] and the whole proposition that follows it are omitted, only implied.

Excerpt (4) is taken from a natural, informal conversation between four female participants (all under 25, university students).

(4)

[KIP, TOA3012]

TO091:  *non lo so*, *io non l’ho sentita*::, *quand’ è l’ ultima volta che l’ho sentita*?

    I don’t know, I haven’t heard from her, when did I last hear from her?

TO091:  *boh*

    dunno

TO085:  *°che ne so io*?*°*

    °how should I know?°

TO091:  ***mi sa*** *quando c’eri pure tu*, *[…]*

I guess when you were there too, […]

Participants are talking about a common acquaintance, a girl that TO091 hasn’t heard from in a while. She asks a question mainly addressed to herself (“when did I last hear from her?”), in an attempt to remember, as TO085’s answer seems to emphasise (How should I know something concerning your personal area of experience?). It is interesting to note that, on the one hand, TO091’s question, although produced in interaction, appears to be self-addressed and, on the other hand, TO085’s answer has a similar character, both because of its redundancy (it is apparent that the content is beyond her “territory of information” [55]) and since it is pronounced with a lower volume (as if speaking to herself). Both interlocutors here, respectively with a wh-question and an epistemic disclaimer, express an unknowing position.

In her following turn, TO091 seems to recall the information and answers her own question, but she exposes it as something of which it is not fully certain (*mi sa*). In this case, *mi sa* is inserted in an elliptical proposition that, if completed, could sound as: *mi sa (che l’ultima volta che l’ho sentita è stata) quando c’eri pure tu* [I think (that the last time I heard from her was) when you were there too]. In the occurrences of this type, usually it is just the completive proposition introduced by *che* [that] which is implied. In doing this, she shifts from an unknowing to an uncertain, believing position.

*Mi sa di*. The following excerpt is taken from the same conversation as (4).

(5)

[KIP, TOA3012]

TO085:  *ma come sono le*::, *stanze in residenza*?

    but how are the rooms in the residence?

TO091:  *fighe*::, *ma lui c’ha ’na [stanza*,]

    cool, but he’s got a [room,]

TO085:        *[perché]* ***mi sa tanto di***:: *collegio*

         [because] to me it looks a lot like a boarding school

TO090:  *sì sembra ‘n ospedale psichiatrico come [abbiam detto ieri con Marta*,]

     yes it looks like a psychiatric hospital as [we said yesterday with Marta,]

TO085:           *[eh esatto]*

              [eh that’s right]

The three participants are talking about the rooms in the university residence. TO085 asks a question about their quality and TO091’s answer (“cool”) seems to disconfirm her impression or pre-judgment. TO085’s question is introduced by the negative conjunction *ma* [but], implying a dubitative stance, and followed, in her subsequent turn, by the reason for the question itself: *perché* (la residenza) *mi sa tanto di collegio* [because to me it (the residence) looks a lot like a boarding school]. In other words, TO085 seems to ask TO091 (who has seen the rooms) a confirmation of her subjective impression: the external appearance of the building, which recalls a boarding school, leads her to imagine that the rooms are not so nice. TO085’s impression is endorsed by TO090, the third participant to the conversation, who reinforce it, even comparing the building to a psychiatric hospital. TO085 fully agrees with her (“eh that’s right”).

Here the expression *mi sa*, although still used to express a subjective (*mi* [to me]) belief, opinion, impression, supposition, etc., seems to maintain the connection with the original meaning of *sapere* as ‘to have taste’, more than with that of ‘to know’. This expression *mi sa di* [literally: to me it tastes of, that we can translate with ‘to me it looks/smells/sound like’] is typically used by the speaker to communicate that they perceive a similarity between two objects, facts or events; literally, the things compared have the same taste. The processes involved are both perceptual and cognitive (memory, imagination, etc.) in nature. Sometimes, in the spoken Italian, boosting or mitigating adverbs can be inserted in the expression *mi sa di*: for example *tanto* [a lot], as in the excerpt (5) (*mi sa tanto di* [to me it looks a lot like]), and *un po’* [a bit] (*mi sa un po’ di* [to me it looks a bit like]).

Reflecting on the occurrences of *mi sa di*, we realised that such expression, differently from *mi sa che + proposition*, has no morphological restrictions (see the Introduction): it can be referred to any person, not only the first one, and used with any tense, not only the simple present, so that all the following sentences sound grammatically correct in Italian:

*Questo posto mi sa/a me (ci/a noi) sa (sapeva*, *saprà) di collegio*

[This place looks (looked, will look) like a college to me (us)]

*Questo posto ti/a te (vi/a voi) sa (sapeva*, *saprà) di collegio*

[This place looks (looked, will look) like a college to you]

*Questo posto a lei/lui (a loro) sa (sapeva*, *saprà) di collegio*

[This place looks (looked, will look) like a college to her/him (them)].

*Mi sa di no*. However, we must note the case in which mi sa di can also take on the meaning of I believe/think/suppose/imagine (thus, analogous to mi sa che), i.e., when it is followed by the affirmative or negative adverbs sì [yes] and no [no], as in the following example:

(6)

[ParlaTO, PTD019]

TOR001:  *va bene ehm*:: *(beh) la domanda dopo era hai frequentato le scuole nel quartiere*

     okay um (well) the next question was did you attend schools in the neighbourhood

TOR001:  *in cui vivi ma* ***mi sa di no***:?

     you live in but I guess not?

TOI011:   *no*.

     no.

TOR001:  *no vero*?

     no right?

TOR001:  *okay*.

     okay

The above excerpt is taken from a semi-structured interview, about ‘home, work and hobbies’, among three participants, one female (interviewer) and two males (interviewees) (age range 26–30). The interviewer (TOR001) asks a question, anticipating the possible (negative) answer and somehow conveying her epistemic access thanks to *mi sa*: as a matter of fact, she makes a hypothesis based on inference, since the interviewee has previously said (the passage is not reported in this sequence) that he is from a city different from the one where he lives now.

The epistemic dynamic embedded in TOR001’s utterance is very similar to that illustrated by commenting the example (3): in the same turn, she shifts from a Not Knowing Whether (expressed by the yes/no question) to a believing position (conveyed by *mi sa di no*), with a kind of self-correction, preceded by “but”.

*Mi sa parenthetical*. In its parenthetical use, *mi sa* has been found in the corpus both in medial and in final position.

*Medial position*: the following excerpt is taken from a semi-structured one-to-one interview, between two female participants, under 25. The main topic of the interview is the wide concept of ‘home’.

(7)

[KIP, TOD2016]

TO071:  *faccio un altro paio di domande*,

     I ask a couple more questions,

TO071:  *allora*

     so

TO071:  *ti chiedo come ultime domandine*,

     I ask you as last little questions,

TO071:  *di raccontarmi degli aneddoti*.

     to tell me some anecdotes.

TO071:  *okay*?

     okay?

[…]

TO080:  *va bene anche sui vicini*?

     is it also ok about the neighbours?

TO071:  *assolutamente*

     absolutely

TO080:  *perché*:,

     because,

TO080:  *io e la mia famiglia*,

     me and my family,

TO080:  *la sfiga coi vicini ce l’abbiamo* ***mi sa*** *nel DNA*,

     bad luck with neighbours is I guess in our DNA,

TO071:  *okay*

     Okay

The interviewer TO071 asks TO080 to tell some anecdotes; the interviewee asks if the anecdote can concern her neighbours and, after TO071’s full expression of agreement, she starts her narration, anticipated by a consideration: both she and her family have always been unlucky with their neighbours. To express this concept, the speaker uses a hyperbolic metaphorical expression (“bad luck with neighbours is in our DNA”), whose aspect of exaggeration seems to be mitigated by the parenthetic *mi sa* (that here can be translated as I guess, I have the impression/feeling, etc.), expressing a Believing position.

*Final position*: this last excerpt is taken from the same conversations as (4). Participants are talking about this and that, shifting freely from one topic to another. In this short fragment, they are talking about two great Italian authors of the 14th century (Dante Alighieri and Giovanni Boccaccio), who are studied in high school.

(8)

[KIP, BOA3001]

BO003:  *però sì*. *Dante è quello più bello da fare che Boccac[cio >sec-< quello è bello]*

     but yes. Dante is nicer to study than Boccac[cio >sec-< that’s nice]

BO002:           *[sì (*.*)] a me Boccaccio non piace*.

           [yes (.)] I don’t like Boccaccio.

BO002:  *sarà che (*.*) no*. *ma me l’hanno fatto odiare al lice*:*o* ***mi sa***

     maybe (.) no. but they made me hate him in high school I guess.

BO002, after stating that she does not like Boccaccio, also gives a possible reason for this. She first uses the epistemic future *sarà* [lit.: it will be, that can be better translated with ‘maybe’]), introducing a supposition, but then she stops, makes a brief pause followed by a *no* [no], as if she wanted to correct what she is about to say; then she rephrases her supposition by using *mi sa* in the final position: she imagines, has the impression, supposes that her high school teachers (“they”) made her hate Boccaccio. From an epistemic perspective, the speaker conveys a believing position, based on memory as evidential access.

On closer examination, the six types of structures into which the occurrences of *mi sa* have been subdivided could be basically reduced to two: *mi sa che + p* and *mi sa di* (metaphorical).

The structures *mi sa che…(pending)*, but also *mi sa + elliptical p* and *mi sa parenthetical* implicitly refer to a *mi sa che + p* structure:

1. mi sa + elliptical p (example 4): ***mi sa*** *quando c’eri pure tu* [I guess when you were there too] = ***mi sa che*** *(l’ultima volta che l’ho sentita è stata) quando c’eri pure tu* [I think (that the last time I heard from her was) when you were there too;
2. mi sa parenthetical (example 8): *me l’hanno fatto odiare al liceo* ***mi sa*** [they made me hate him in high school I guess] = **mi sa che** me l’hanno fatto odiare al liceo [I guess that they made me hate him in high school].

In *Mi sa di no/(sì)* [I think not/(yes)], the adverbs *no/(sì)* [no/(yes)] refer to the truth value of an implicit p (example 6: *Mi sa di no* [I think not] = *Mi sa che non hai frequentato le scuole nel quartiere in cui vivi* [I think that you did not attend schools in the neighbourhood you live in]), so it could also be seen as an elliptical expression.

These four types of structures are different in morphology from each other and from the basic structure *mi sa che + p*, but their underlying meaning is the same. Thus, the only two types of structures that differ in meaning (and morphology, of course) seem to be *mi sa che + p* and *mi sa di (metaphorical)*.

In the Introduction, we saw that, following Serianni [38] (p.19) and Cialdini [37], the verb of the proposition introduced by *mi sa che* is normally in the indicative mood, seldom in the conjunctive. In this regard (indicative or conjunctive?), which are the data coming from the KIParla spoken corpus?

We made a grammatical analysis of all the propositions introduced by *mi sa che* in order to find out not only which is the most frequent mood but also which are the most used tenses and subjects (first, second, third person). As a result, no conjunctive mood was found, only indicative. The most used tense is the simple present. The most frequent subject is the third person singular, as for example

(9) [KIP, B0A3001

B0OO2:  *nel primo libro mi sa che arriva solo alla fine*

      in the first book I think that he arrives only at the end

where the completive proposition has the third person singular as its subject and the verb in the simple present.

These grammatical data helped us in the creation of the completive proposition *Andrea sta andando a Verona* [Andrea is going to Verona] that we used for the questionnaire in Study 2 (see the beginning of the next section).

## Study 2: The epistemic relationship of *mi sa* with *so* [I know], *non so* [I do not know], *non so se* [I do not know whether], *credo* [I believe], *penso* [I think]

Research questions 2–4 (section Aims) aimed to explore the epistemic relationships of *mi sa* with *so* [I know], *non so* [I do not know], *non so se* [I do not know whether], *credo* [I believe] and *penso* [I think].

In order to answer these questions, a sample of Italian speakers was administered a questionnaire comparing 6 sentences, in which the same propositional content (*Andrea is going to Verona*) is introduced by the above mentioned six modal expressions (See Supporting information for the questionnaire in its English translation).

As anticipated at the end of the previous section, such completive proposition was created *ad hoc* for the questionnaire, primarily on the basis of the grammatical data resulting from the spoken corpus (the proposition should have the third person singular as its subject and a verb in the simple present); secondly, the propositional content should obviously fit syntactically and semantically the above mentioned six modal expressions. That is why, among many (almost infinite) possible contents, we chose *Andrea sta andando a Verona* [Andrea is going to Verona], where the present continuous *sta andando* [is going] fits the six modal expressions better than the simple present *va* [goes].

The structure of the questionnaire is based on the theoretical framework presented in a previous section. In particular, the questionnaire will test our suppositions about the epistemic relationships among the six markers.

### Methods and materials

#### Data collection

This study, conducted according to the APA Ethics Code (https://www.apa.org/ethics/code, last accessed 17th of March 2022), and European and Italian Privacy Law (i.e., EU Reg. 679/2016, GDPR and Legislative Decree n. 196/2003, Code regarding the protection of personal data), has been approved by the Ethic Committee of the University of Macerata (March 28^th^ 2022).

It was conducted through an online survey, that was available online from June 15 to September 24 2021. The questionnaire was initially proposed not only via email, but also through social media (e.g. Facebook and WhatsApp), a context which is certainly not representative of the entire population, but through which different types of individuals can be reached. Many of these ‘different individuals’ initiated the typical ‘word of mouth’ of the snowball sampling with the outcome of having generated not one, but several snowball samples. Our sample is a set of snowball samples, which is why we believe it is adequately representative of the population. Survey administration was conducted through LimeSurvey software (version 3.22; [56]) on a LAMP (Linux, Apache, MySQL, PHP) web-server. The HTTPS protocol and secure sockets layer (SSL) were used to encrypt all traffic. Inclusion criteria: adults ≥ 18; exclusion criteria: subjects < 18.

The questionnaire, titled *La comunicazione di ciò che conosciamo*, *non conosciamo o di cui siamo incerti* [The communication of what we know, we do not know or we are uncertain about], opened with some information concerning the aims of the research, the identity of the research team, the planned ways of disseminating the results, the references to the European and Italian privacy laws, and protection of personal data. The respondents could begin to fill in the questionnaire after having voluntarily consented to participate, by signing an online informed consent.

In the first part of the questionnaire, the participants had to answer 4 socio-demographic questions; in the second one, they had to assign an epistemic meta-paraphrase to 6 sentences in which the same propositional content (p = *Andrea is going to Verona*) was introduced by a different epistemic expression (see section First task). Finally, they had to assign a degree of uncertainty, by using a scale that goes from 1 (*very little uncertainty*) to 10 (*very much uncertainty*), to those sentences for which they chose the uncertain meta-paraphrase (see section Second task).

All the items of the questionnaire were compulsory. The estimated average time for compiling the questionnaire was approximately 10 min.

#### Measures

*The questionnaire*: *The communication of what we know*, *we do not know or we are uncertain about*. As anticipated, the questionnaire compares 6 sentences, in which the same proposition p (= *Andrea is going to Verona*) is introduced by a different epistemic marker:

1. *so che p* [I know that p]
2. *non so perché p* [I do not know why p]
3. *non so se p* [I do not know whether p]
4. *mi sa che p* [to me it knows that p]
5. *credo che p* [I believe that p]
6. *penso che p* [I think that p]

The constant is the propositional content p (*Andrea is going to Verona*), the variables are the six epistemic markers.

Participants are invited to accomplish the following two tasks.

*First task*. The 6 sentences are presented randomly to each participant and one at a time, not all together, since after each sentence the participants have a first task to accomplish, i.e., to choose among the following three possible *epistemic meta-paraphrases*, i.e., metalinguistic descriptions, the one that in their opinion is the nearest to the sentence at issue:

1. *Il parlante comunica di* ***essere certo*** *che Andrea stia andando a Verona* [The speaker communicates **to be certain** that Andrea is going to Verona]
2. *Il parlante comunica di* ***non essere certo*** *che Andrea stia andando a Verona* [The speaker communicates **not to be certain** that Andrea is going to Verona]
3. *Il parlante comunica di* ***non essere a conoscenza*** *del fatto che Andrea stia andando a Verona* [The speaker communicates **not to know** that Andrea is going to Verona]

Such *epistemic meta-paraphrases*, from now on abbreviated as EMPs, compare three different *epistemic positions* of the speaker: *knowing*, *uncertain*, *unknowing*.

As for the unknowledge EMP (c) *Il parlante comunica di* ***non essere a conoscenza*** *del fatto che Andrea stia andando a Verona* [The speaker communicates **not to know** that Andrea is going to Verona], we are fully aware of the fact that we cannot say *Non so ***che*** *Andrea sta andando a Verona* [*I do not know that Andrea is going to Verona]: this sentence is unacceptable for the following reasons.

In the Introduction (example 1), we claimed that the sentence *So che p* [I know that p] presupposes that p is true. Such a presupposition is part of the speaker’s *background knowledge*. If they say *Non so che p [*I do not know that p], on the one hand, they say that p is part of their background knowledge, i.e., that they know that p; on the other hand, with the same sentence they deny that it is so. There is thus a contradiction in what they say. It is to this contradiction that the unacceptability of *Non so che p [*I do not know that p] is due.

On the contrary, there is no contradiction in the sentence ***Non sapevo che*** *p* [**I did not know that** p] (which implies that now I know that p) as well as in the sentence **Lei/Lui non sa che** p [**She/He does not** know that p], i.e., when the subject of the sentence is different from the speaker’s ‘I’. In this latter case, the sentence presupposes that p is true, but it refers to another person, a third person, not to the speaker themselves, as instead it happens in the sentence *Non so che p [*I do not know that p]: it is that third person, not the speaker themselves, who does not know that p [57–59].

Thus, in our opinion, the unknowledge EMP (c) *Il parlante comunica di* ***non essere a conoscenza*** *del fatto che Andrea stia andando a Verona* [The speaker communicates **not to know** that Andrea is going to Verona] and the sentence *Lei/Lui non sa che Andrea sta andando a Verona* [She/He does not know that Andrea is going to Verona] are semantically similar and the former can be taken as a possible EMP of the latter.

In Study 2, our purpose concerning the three EMPs was to understand which epistemic position, in the participants’ opinion, the speaker communicates in each of the six sentences: knowing, uncertain, unknowing. In this context, the unknowledge EMP (c) seemed to us to be one of the very few possible EMPs consistent with the unknowing position, i.e., able to give participants an (intuitive) idea of such position.

From a syntactic and semantic point of view, the three EMPs fit sentences (1) and (3)-(6) but do not fit sentence (2), due to the presence of *why*; so the EMPs were changed a little in the following way (see the words underlined):

(a) *Il parlante comunica di* ***essere certo*** *riguardo ai motivi per cui* *Andrea stia andando a Verona* [The speaker communicates **to be certain** about the reasons why Andrea is going to Verona]
(b) *Il parlante comunica di* ***non essere certo*** *riguardo ai motivi per cui* *Andrea stia andando a Verona* [The speaker communicates **not to be certain** about the reasons why Andrea is going to Verona]
(c) *Il parlante comunica di* ***non essere a conoscenza*** *dei motivi per cui* *Andrea stia andando a Verona* [The speaker communicates **not to know** the reasons why Andrea is going to Verona].

*Expectations about the first task*. In accordance with the theoretical framework, participants are expected to choose:

- the certainty EMP (a) for sentence (1) *so che p* [I know that p];
- the unknowledge EMP (c) for (2) *non so perché p* [I do not know why p];
- the uncertainty EMP (b) for (3) *non so se p* [I do not know whether p], (4) *mi sa che p* [To me it knows that p], (5) *credo che p* [I believe that p], and (6) *penso che p* [I think that p].

*Second task*. If, and only if, a participant chooses the uncertainty EMP for the sentence at stake (independently from which sentence is at stake), they are invited to accomplish a second task: to evaluate how much uncertainty the sentence communicates by using a scale that goes from 1 (*very little uncertainty*) to 10 (*very much uncertainty*). Should not a participant choose the uncertainty EMP, they would proceed with the next sentence, i.e., they are not requested to evaluate the degree of uncertainty of the sentence at stake if they have not chosen the uncertainty EMP.

*Expectations about the second task*. Participants are expected to assign sentences (4) *mi sa* / (5) *credo* / (6) *penso che p* about the same degree of uncertainty, thus confirming that they are synonyms.

Sentence (3) *non so se p* is expected to be assigned a different degree of uncertainty, higher than the one assigned to (4) *mi sa* / (5) *credo* / (6) *penso che p*, thus confirming that in the epistemic continuum of uncertainty sentence (3) *non so se p* refers to the pole where uncertainty is higher (*not knowing whether* pole), while sentences (4) *mi sa* / (5) *credo* / (6) *penso che p* refer to the pole where uncertainty is lower (*believing* pole).

*Sample characteristics*. The questionnaire was filled in by 201 participants.

About two thirds of the participants are female (157, 78.11%) and have a university level of education (BA: 17.91% + MA: 23.38% + Post graduated: 29.35% = 70.64%); about one third has a pre-university level of education (Middle school diploma: 4.98% + High school diploma: 24.38% = 29.26%). Almost all participants are Italian native speakers (92.04%; 4.48% are bilingual; only 3.48% are not Italian mother-tongue) and their age ranges from 18 to 75 years (mean age 43.48).

### Results

#### First part of the questionnaire: Which EMPs the participants chose for which sentences

**Fig 3:**
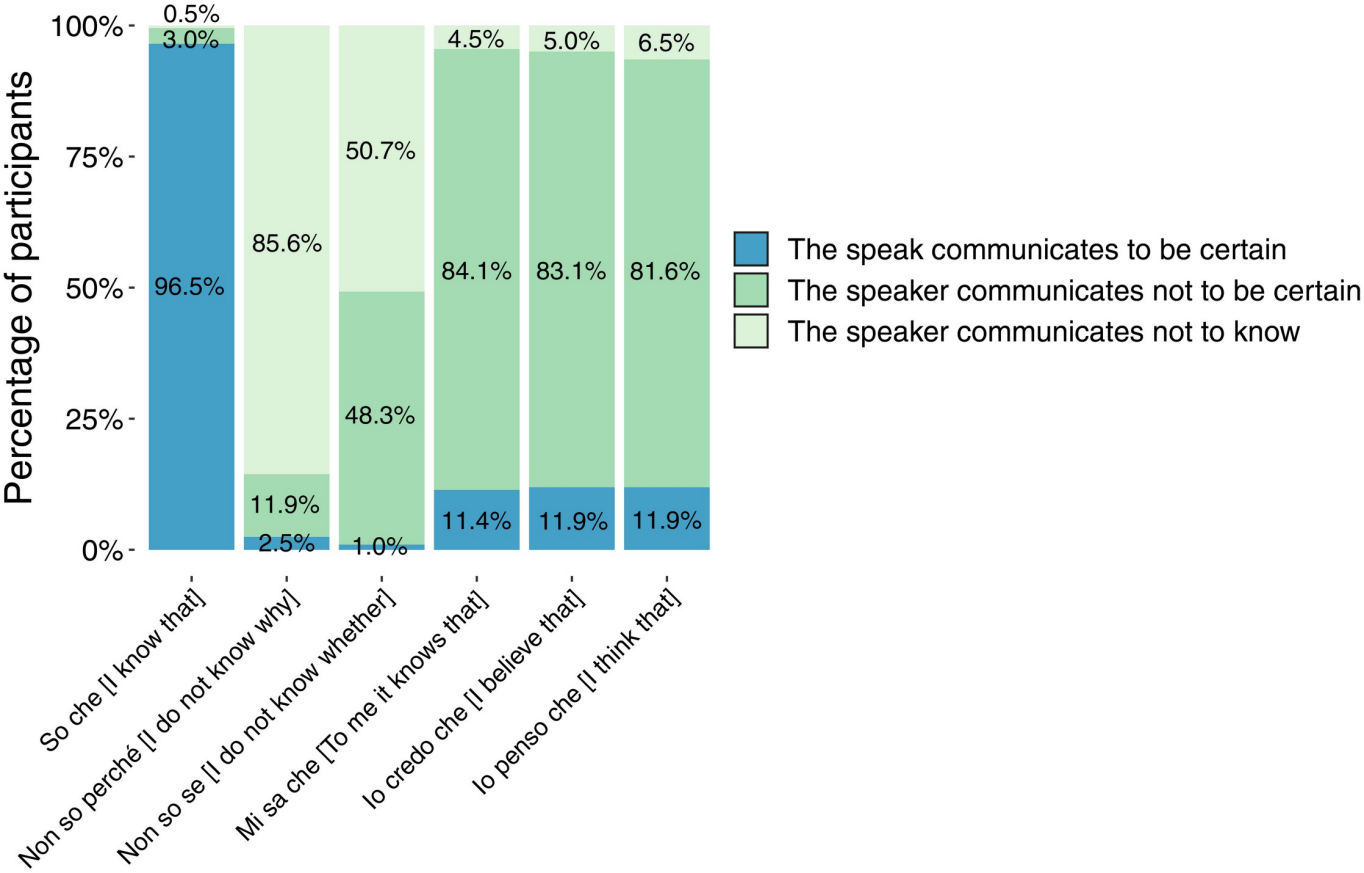
Percentages, rounded to the first decimal, of participants who chose which EMPs for which sentences.

Table 2 and Fig 3 show the quantitative results of the first part of the questionnaire.

**Table 2:** Numbers and percentages of participants who chose which EMPs for which sentences.

	(a)The speaker communicates to be certain	(b)The speaker communicates not to be certain	(c)The speaker communicates not to know	Tot.
***(1) So che* [I know that]** *Andrea is going to Verona*	194 *(96*.*52%)*	6 *(2*.*99%)*	1 *(0*.*50%)*	201 *(100%)*
***(2) Non so perché* [I do not know why]** *Andrea is going to Verona*	5 *(2*.*49%)*	24 *(11*.*94%)*	172 *(85*.*57%)*	201 *(100%)*
***(3) Non so se* [I do not know whether]** *Andrea is going to Verona*	2 *(1%)*	97 *(48*.*25%)*	102 *(50*.*75%)*	201 *(100%)*
***(4) Mi sa che* [To me it knows that]** *Andrea is going to Verona*	23 *(11*.*44%)*	169 *(84*.*08%)*	9 *(4*.*48%)*	201 *(100%)*
***(5) Credo che* [I believe that]** *Andrea is going to Verona*	24 *(11*.*94%)*	167 *(83*.*08%)*	10 *(4*.*98%)*	201 *(100%)*
***(6) Penso che* [I think that]** *Andrea is going to Verona*	24 *(11*.*94%)*	164 *(81*.*59%)*	13 *(6*.*47%)*	201 *(100%)*

Table 2 shows the number and percentages of participants who chose which EMP for which sentence. Fig 3 shows only the percentages, rounded to the first decimal.

Table 2 shows that for sentence (1) *So che p* [I know that p], 96.52% of participants chose the certainty EMP (a) *The speaker communicates to be certain that p*, as expected.

For sentence (2) *Non so perché p* [I do not know why p], 85.57% of participants chose the unknowledge EMP (c) *The speaker communicates not to know the reasons why p*, as expected.

Sentence (3) *Non so se p* [I do not know whether p] was expected to be assigned the uncertainty EMP (b) *The speaker communicates not to be certain that p*. Participants divided into two approximately equal groups: 48.25% chose the uncertainty EMP (b); 50.75% the unknowledge EMP (c) *The speaker communicates not to know that p*.

As expected, sentences (4) *Mi sa che p* [To me it knows that p], (5) *Credo che p* [I believe that p], (6) *Penso che p* [I think that p] were assigned the uncertainty EMP (b) *The speaker communicates not to be certain that p* with almost the same percentages: 84.08%, 83.08%, 81.59%, respectively.

To sum up, all such results are in line with the expectations, except those concerning sentence (3), which will be commented on in the Discussion.

#### Statistical analysis

The original multinomial format of the responses was initially transformed into a binomial format using the ‘mlogit’ package [60] of the R statistical software [61].

A series of Generalized Linear Mixed Models (GLMM) were then performed (one for each sentence/epistemic marker), mainly using R’s lme4 package, setting the ‘family’ parameter to binomial and the ‘link-function’ parameter to logit. The outcome variable was necessarily of type 0/1, where 0 meant ’no response chosen’ and 1 ‘response chosen’. The only fixed factor was the EMP variable with its 3 levels. The only random factor was the subject ID. The Analysis of Deviance (Wald chi square tests) was used as omnibus test. We used the Bonferroni correction for post-hoc tests (emmeans R package, [62]).

This statistical analysis was chosen because it allows the distribution of dichotomous responses to be studied while respecting statistical assumptions and taking into account the fact that each subject provides more than one response. Much literature exists on the use of this procedure. For a systematic review see [63].

We previously tested (using the The Akaike Information Criterion) whether the addition of demographic variables such as ‘age’ and ‘education level’ (and their interaction) to the reported analysis model could improve the fit of the model. This never happened. The model that does not consider these variables as covariates is always the best fit.

#### Sentence (1) *So che p* [I know that p], certainty EMP (a)

**Fig 4:**
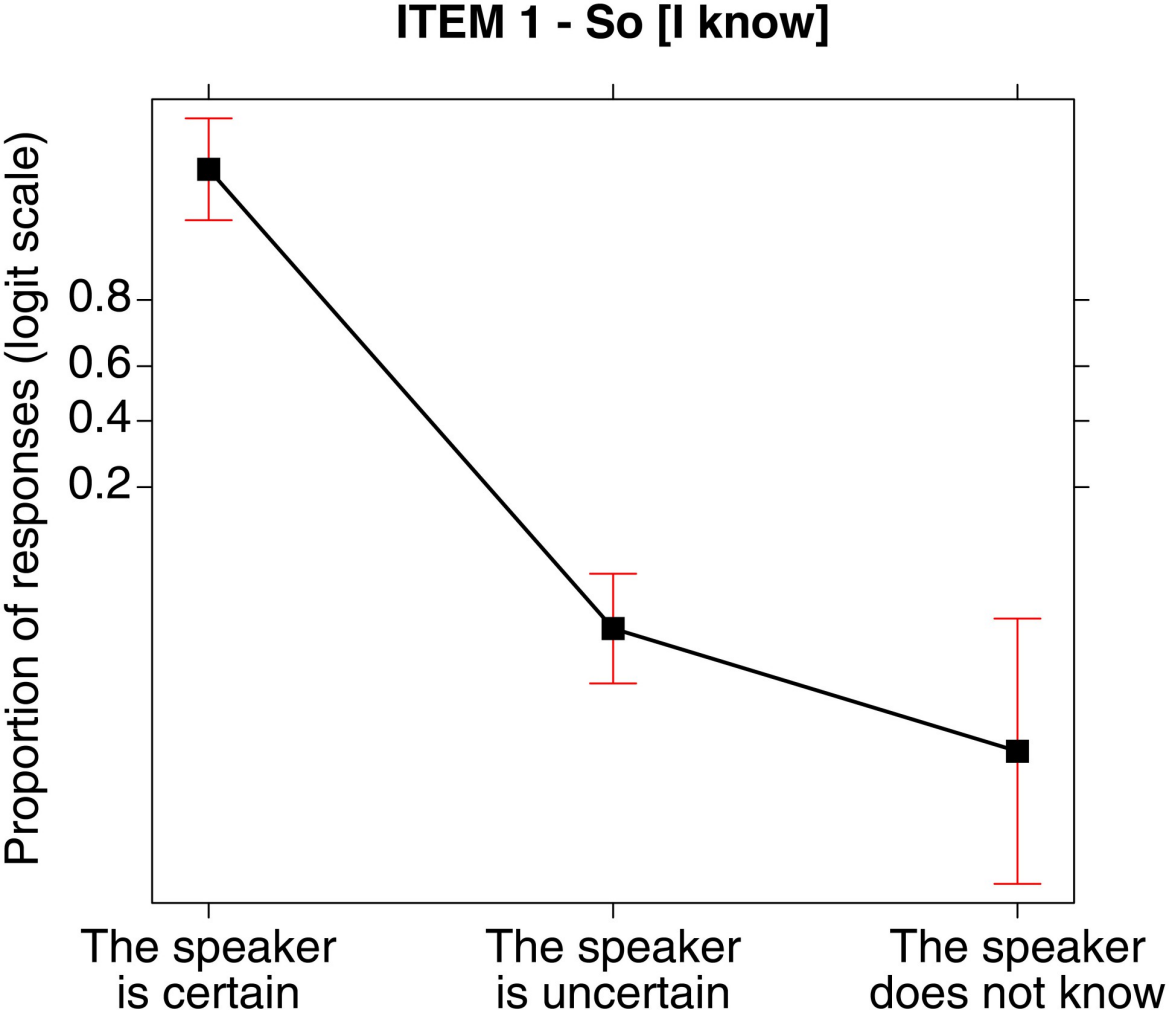
Sentence (1) *So che p* [I know that p] and the three EMPs: Probability of choosing one of them for such sentence.

As for sentence (1), the results of the analysis revealed statistically significant differences χ^2^ (2, N = 201) = 172.340, p < .001 concerning the EMPs selected by the participants. Specifically, the results of the analysis pointed out not only that the certainty EMP (a) was the one preferred by the participants, but also that this choice significantly differs from both the unknowledge EMP (c) (post-hoc: z = 8.028, p < .001), and the uncertainty EMP (b) (post-hoc: z = 12.030, p < .001) respectively, as it is clearly visible also from the following Fig 4. Vice versa, no significant difference was found between EMPs (b) and (c) (post-hoc: z = 1.675, p = .281), which were selected by a lower number of participants.

#### Sentence (2) *Non so perché p* [I do not know why p], unknowledge EMP (c)

**Fig 5:**
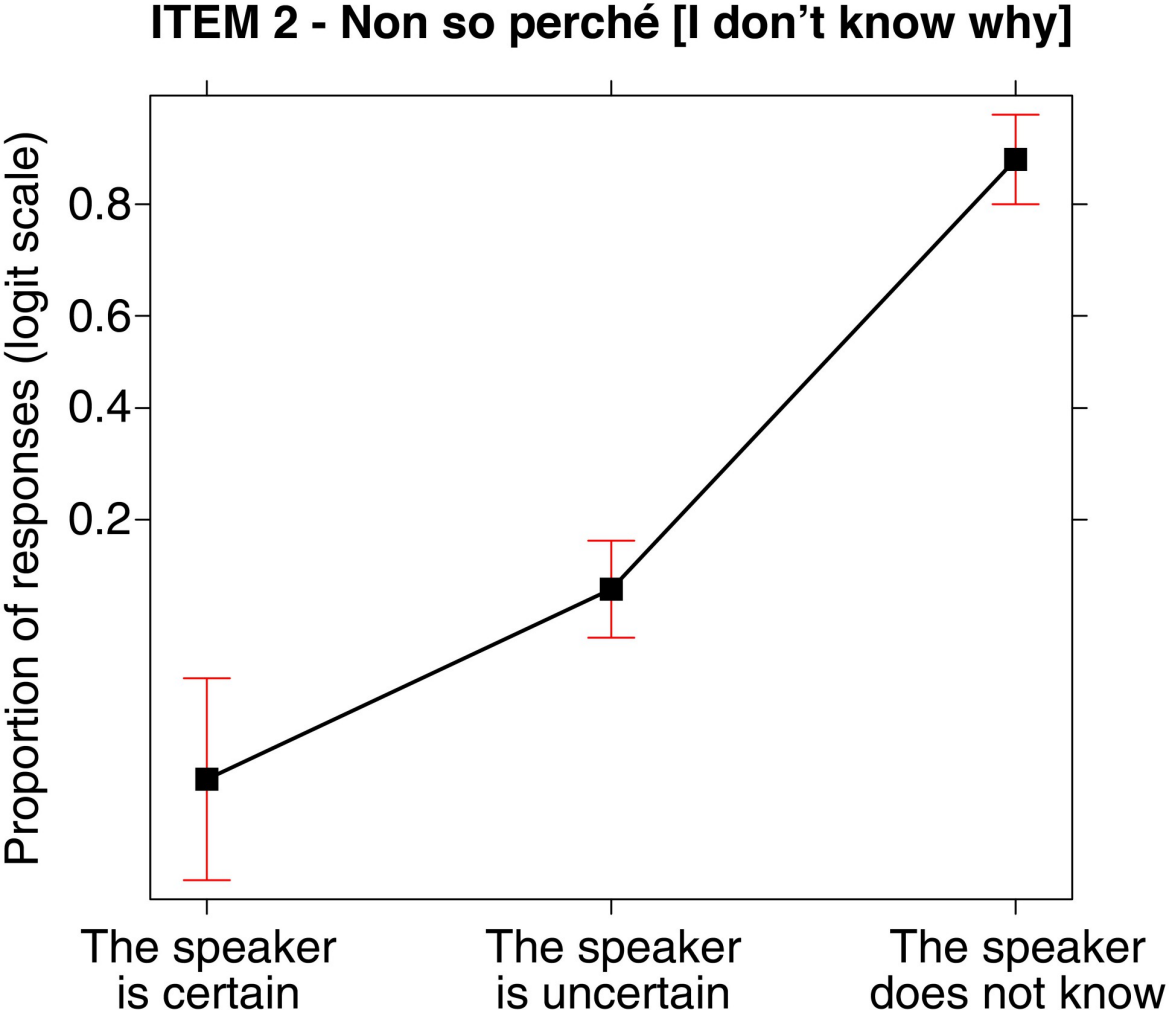
Sentence (2) *Non so perché p* [I do not know why p] and the three EMPs: Probability of choosing one of them for such sentence.

As for sentence (2), the analysis revealed statistically significant differences χ^2^ (2, N = 201) = 223.643, p < .001 concerning the EMPs selected by the participants. In particular, the analysis pointed out not only that the unknowledge EMP (c) was chosen by the majority of participants, but also that it significantly differs from the other two, i.e., both from the certainty EMP (a) (post-hoc: z = -10.999, p < .001) and the uncertainty EMP (b) (post-hoc: z = -12.765, p < .001) respectively. The analysis also revealed significant differences between the choice of the certainty EMP (a) and the uncertainty EMP (b) (post-hoc: z = -3.325, p = .003), see Fig 5:

#### Sentence (3) *Non so se p* [I do not know whether p], uncertainty EMP (b) and unknowledge EMP (c)

As for sentence (3), the analysis revealed significant differences χ^2^ (2, N = 201) = 40.986, p < .001 concerning the EMPs selected by the participants. Specifically, although the results of the analysis did not reveal significant differences between EMPs (b) and (c) (post-hoc: z = -0.499, p = 1.00)–which were the most selected–, conversely, it pointed out significant differences between EMPs (a) and (c) (post-hoc: z = -4.630, p < .001) and between EMPs (a) and (b) (post-hoc: z = -4.531, p < .001).

#### Sentences (4) *Mi sa che p* [To me it knows that p], uncertainty EMP (b)

As for sentence (4), the analysis revealed highly significant statistical differences χ^2^ (2, N = 201) = 230.668, p < .001 concerning the EMPs selected by the participants. Specifically, the results of the analysis showed that the uncertainty EMP (b) was the most selected, and that it significantly differs from the choice of both the certainty EMP (a) (post-hoc: z = 12.633, p < .001) and the unknowledge EMP (c) (post-hoc: z = 12.059, p < .001), which were less selected. Significant differences were also identified between the certainty EMP (a) and the unknowledge EMP (c) (post-hoc: z = 12.059, p < .001).

#### Sentence (5) *Credo che p* [I believe that p], uncertainty EMP (b)

As for sentence (5), the analysis revealed highly significant statistical differences χ^2^ (2, N = 201) = 227.753, p < .001 concerning the EMPs selected by the participants. Specifically, the results of the analysis pointed out that the uncertainty EMP (b) was the most selected, and that it significantly differs from both the certainty EMP (a) (post-hoc: z = 12.110, p < .001) and the unknowledge EMP (c) (post-hoc: z = 12.482, p < .001), which were less chosen. Significant differences were also identified between the certainty EMP (a) and the unknowledge EMP (c) (post-hoc: z = 2.436, p = .044).

#### Sentence (6) *Penso che p* [I think that p], uncertainty EMP (b)

As for sentence (6) the analysis revealed highly significant statistical differences χ^2^ (2, N = 201) = 224.132, p < .001 concerning the EMPs selected by the participants. Specifically, the results of the analysis revealed that the uncertainty EMP (b) was the one preferred by the participants and that this choice significantly differs from both the choice of the certainty EMP (a) (post-hoc: z = 12.295, p < .001) and the unknowledge EMP (c) (post-hoc: z = 12.249, p < .001), which were the least selected. Differently from what has been registered for sentence (4) *Mi sa che Andrea sta andando a Verona* [To me it knows that Andrea is going to Verona] and sentence (5) *Credo che Andrea stia andando a Verona* [I believe that Andrea is going to Verona] no significant differences were identified between the certainty EMP (a) and the unknowledge EMP (c).

**Fig 6:**
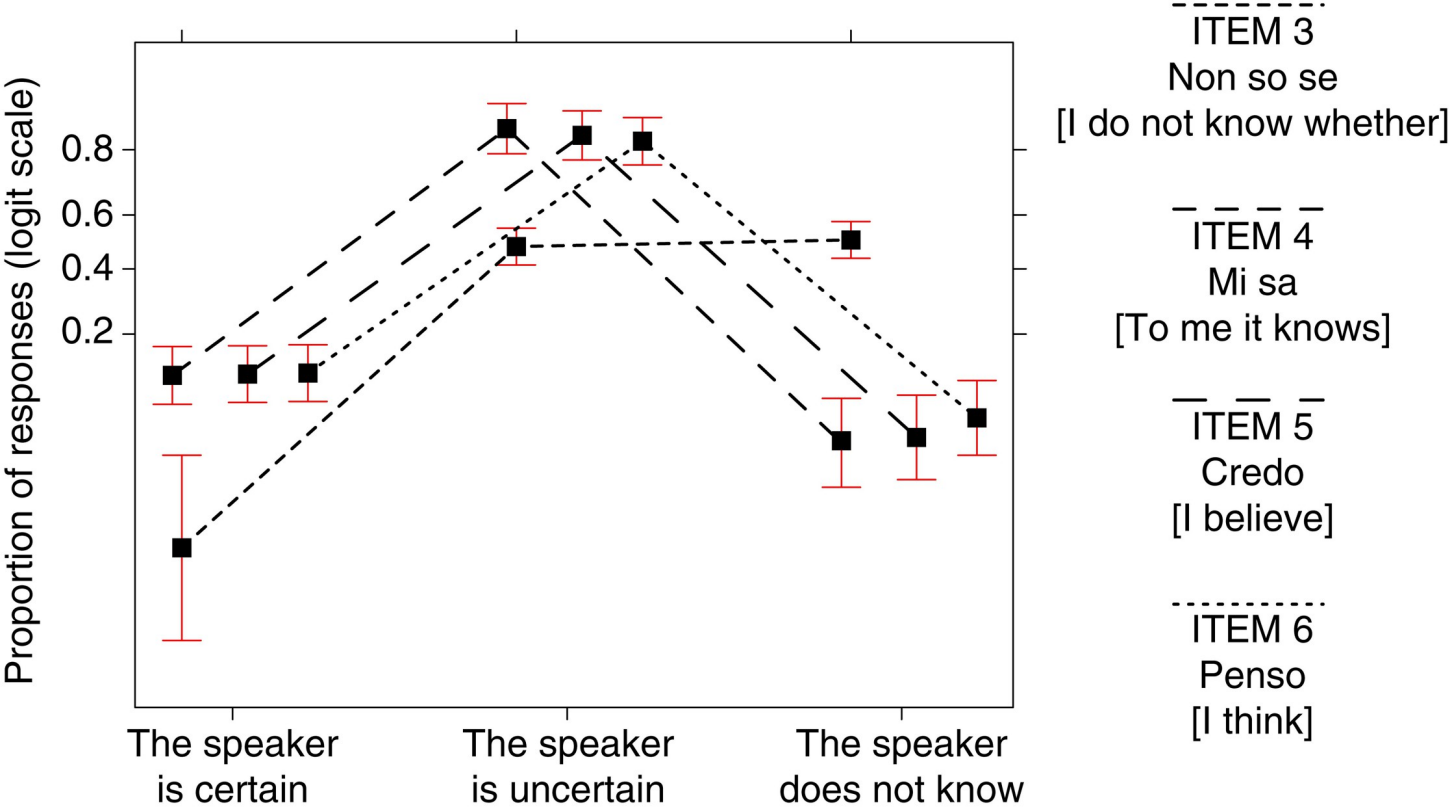
Synoptic view of the proportion of responses for sentences (3), (4), (5), (6) concerning the three EMPs.

The following Fig 6 synoptically represents the proportions of responses concerning the three EMPs for sentences (3), (4), (5), (6).

Therefore, the GLMM reveals that the three sentences (4) *Mi sa che p*, (5) *Credo che p*, (6) *Penso che p*, are thought of by the participants as communicating the speaker’s uncertain epistemic position, specifically the *believing* pole. Nonetheless, the statistical analysis does not reveal if the three markers are synonyms or not. Similarly, it does not disclose if sentence (3) *Non so se p*, on the one side, and sentences (4) *Mi sa che p*, (5) *Credo che p*, (6) *Penso che p*, on the other, place themselves in different points along the uncertain epistemic continuum or not (see Fig 1). To know that, we need to see the results of the second part of the questionnaire.

#### Second part of the questionnaire: Degrees of uncertainty assigned to sentences (3)-(6)

Although the results of the analysis (see Table 2 and Fig 3, as well as Figs 4–6) show that a low percentage of participants—contrary to our expectations—assigned the uncertain EMP to sentences (1) and (2), a higher percentage of them, in line with our expectations, chose the uncertain EMP for sentences (3), (4), (5) and (6).

**Fig 7:**
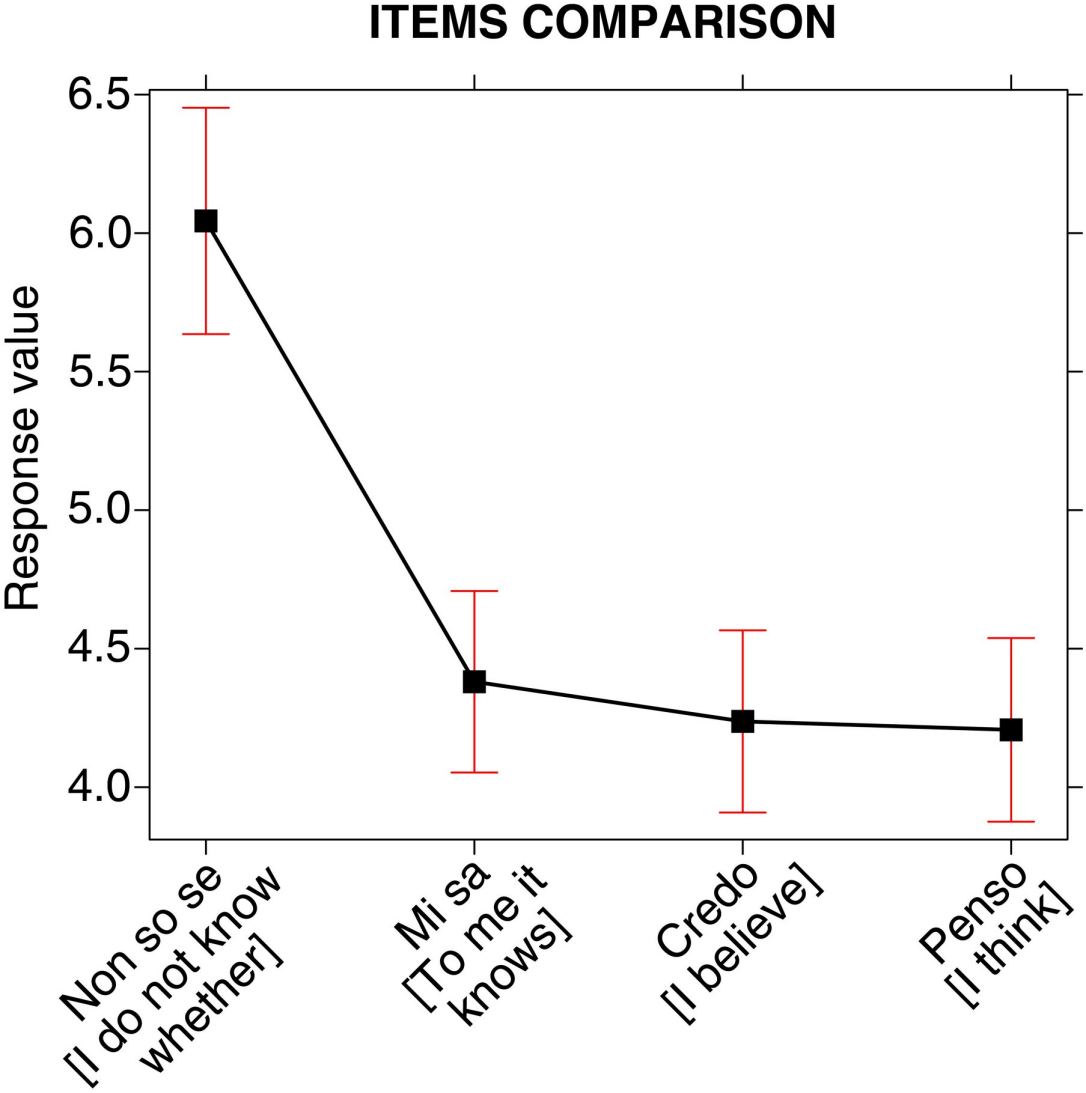
Distribution of the four sentences for degrees of uncertainty.

In order to identify how much uncertainty was assigned to sentences (3)-(6) and verify whether there were significant differences in their distribution, Linear Mixed Models (LMM) was carried out. The results of the analysis (Type III Analysis of Variance Table with Satterthwaite’s method) revealed highly significant differences F(5, 471.270) = 21.006, p < 0.001, indicating therefore that the degrees of uncertainty assigned (i.e., the values selected from a scale, ranging from 1, *very little uncertainty*, to 10, *very much uncertainty*, to the four sentences are different and that it is possible to identify their distribution (in terms of uncertainty) along a gradient. The following Fig 7, which on the vertical axis shows the mean values of each of the four sentences, clearly displays how similar sentences (4), (5) and (6) are, in terms of the uncertainty communicated by the speaker, and how they differ from sentence (3).

As shown in Table 3, 5.90 (fourth column) is the mean degree of uncertainty that 97 participants (second column) assigned to sentence (3) *Non so se Andrea sta andando a Verona* [I do not know whether Andrea is going to Verona].

**Table 3:** The mean values of uncertainty assigned to sentences (3), (4), (5) and (6).

Items	N	Missing	Mean	Median	SD	Minimum	Maximum
3 *non so se*	97	104	5.90	6	2.37	1	10
4 *mi sa che*	169	32	4.39	5	2.04	1	9
5 *credo che*	167	34	4.29	5	2.25	1	10
6 *penso che*	164	37	4.20	5	2.21	1	10

4.39, 4.29 and 4.20 respectively are the mean degrees of uncertainty assigned to sentence (4) *Mi sa che Andrea sta andando a Verona* [To me it knows that Andrea is going to Verona] by 169 participants, to sentence (5) *Credo che Andrea stia andando a Verona* [I believe that Andrea is going to Verona] by 167 participants, and to sentence (6) *Penso che Andrea stia andando a Verona* [I think that Andrea is going to Verona] by 164 participants.

Although the number of participants who consider sentence (3) uncertain is lower than the number of those who consider sentences (4), (5) and (6) uncertain, the mean value of the former sentence is higher than the one of the latter three (5.90 vs 4.39, 4.29, and 4.20). In other terms, sentence (3) is rated as more uncertain than sentences (4), (5) and (6), as expected. Furthermore, as shown in Table 3, no one assigned a score of 10 to sentence (4), i.e., no one assesses it as maximally uncertain (the maximum value assigned was indeed 9).

The following Table 4 shows the Bonferroni post-hoc values according to which there are statistically significant differences between sentence (3), on the one hand, and (4) (p value < .001), (5) (p value < .001), and (6) (p value < .001), on the other hand. In other terms, the results of the analysis would confirm the assignment of similar values of uncertainty to sentences (4), (5) and (6), which would therefore be considered synonymous by the participants.

**Table 4:** Bonferroni-post-hoc comparison of the four sentences.

Comparison							
Item		Item	Difference	SE	t	df	p_bonferroni_
3 non so se	-	4 mi sa che	1.665	0.226	7.374	452	< .001
3 non so se	-	5 credo che	1.806	0.227	7.965	455	< .001
3 non so se	-	6 penso che	1.837	0.225	8.152	445	< .001
4 mi sa che	-	5 credo che	0.143	0.186	0.771	416	1.000
4 mi sa che	-	6 penso che	0.174	0.186	0.931	415	1.000
5 credo che	-	6 penso che	0.031	0.187	0.162	418	1.000

Table 5 shows what degrees of uncertainty from 1 to 10 (first column) were assigned to sentences (3) *Non so se p*, (4) *Mi sa che p*, (5) *Credo che p* and (6) *Penso che p* by how many participants (respectively, columns 2, 4, 6, 8).

**Table 5:** Degrees of uncertainty assigned to the sentences (3) *Non so se p*, (4) *Mi sa che p*, (5) *Credo che p*, (6) *Penso che p*.

	Non so se		Mi sa che		Credo che		Penso che	
Degree of Uncertainty	Frequency	%	Frequency	%	Frequency	%	Frequency	%
**1**	9	9.28	**21**	**12.43**	**25**	**14.97**	**26**	**15.85**
**2**	1	1.03	**12**	**7.10**	**13**	**7.78**	**16**	**9.76**
**3**	2	2.06	**23**	**13.61**	**28**	**16.77**	**24**	**14.63**
**4**	6	6.19	**22**	**13.02**	**15**	**8.98**	**15**	**9.15**
**5**	**27**	**27.84**	**47**	**27.81**	**44**	**26.35**	**43**	**26.22**
**6**	**14**	**14.43**	17	10.06	18	10.78	16	9.76
**7**	**12**	**12.37**	16	9.47	12	7.19	12	7.32
**8**	**13**	**13.40**	8	4.73	3	1.80	6	3.66
**9**	6	6.19	3	1.78	4	2.40	4	2.44
**10**	7	7.22	0	0	5	2.99	2	1.22
**Tot**.	97	100	169	100	167	100	164	100

* The highest frequencies and percentages assigned to the different degrees of uncertainty (ranging from 1 to 10) have been formatted in bold type

As for the sentence (3) Non so se p, the numbers in bold in the second and third columns show that the majority of the participants who chose the uncertainty EMP (b) (97 out 201, 48.25%, see Table 2 and Fig 4) assigned to sentence (3) degrees of uncertainty which range from 5 to 8 (66 out of 97 = 68.04%), thus thickening along the uncertainty continuum that goes from a medium degree (5) towards the pole of *very much uncertainty* (10).

Table 5 shows that, differently from sentence (3), sentence (4) *Mi sa che p* as well as sentence (5) *Credo che p* and sentence (6) *Penso che p* were assigned by the greater number of participants who chose the uncertainty EMP (b) (respectively 169 out of 201 = 84.08%; 167 out of 201 = 83.08%; 164 out of 201 = 81.59%, see Table 2 and Fig 3) degrees of uncertainty which range from 1 to 5 (respectively, 125 out of 169 = 73.97%; 125 out of 167 = 74.85%; 124 out of 164 = 75.61%), thus thickening along the uncertainty continuum that goes from the pole of *very little uncertainty* (1) to a medium degree (5).

**Fig 8:**
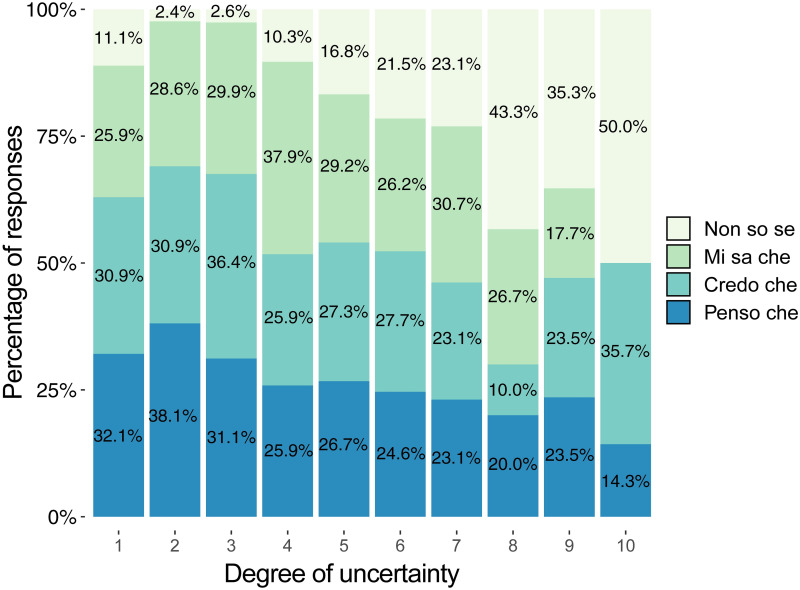
Degrees of uncertainty assigned to the sentences (3) Non so se p, (4) Mi sa che p, (5) Credo che p, (6) Penso che p. *For each degree of uncertainty (for each column) the percentage of *Non so se*, *Mi sa che*, *Credo che* and *Penso che* answers given by the experimental subjects is represented.

The following Fig 8 shows the degrees of uncertainty assigned to the sentences (3) *Non so se p*, (4) *Mi sa che p*, (5) *Credo che p*, (6) *Penso che p*.

Therefore Tables 3–5, and Fig 8 confirm and explain the results shown in Fig 7, where sentence (3) *Non so se p* appears to be evaluated as highly uncertain, i.e., as much more uncertain than sentences (4) *Mi sa che p*, (5) *Credo che p*, (6) *Penso che p* which were given about the same degrees of low uncertainty.

### Discussion

The results achieved through the questionnaire are in line with the expected ones, except those concerning sentence (3) *Non so se p* [I do not know whether p]. Such results need to be discussed.

In the first part of the questionnaire, participants were expected to choose for sentence (3) the uncertainty EMP (b) *The speaker communicates not to be certain that p*. They are divided into two halves: 97, i.e., 48.25% chose the uncertainty EMP (b) *The speaker communicates not to be certain that p*, while 102, i.e., 50.75%, the unknowledge EMP (c) *The speaker communicates not to know that p* (see Table 2 and Fig 3). Such percentages show that for the participants sentence (3) can be thought as communicating *uncertainty* as well as *unknowledge*. If *the majority* of the participants had chosen the unknowledge EMP (c), we would have questioned both our linguistic competence as Italian native speakers and our theoretical model of epistemicity, according to which sentence (3) communicates uncertainty, not unknowledge.

But the above mentioned two *similar* percentages (48.25% and 50.75%) tell us that it is not so. What should be explained is why 50.75% of participants chose the unknowledge EMP. The following hypothetical explanations can be advanced.

First of all, we are aware of the fact that the EMP (c), by which we tried to express the unknowing position (as anticipated in the First task section) has an intrinsic complexity that can make its interpretation ambiguous for participants (the speaker communicates that they do not know something that is asserted as a fact, namely “Andrea is going to Verona”), such that a partial overlap with the EMP (b) can be justified on a superficial reading.

The choice of the unknowledge EMP (c) for sentence (3) could be also due to the fact that, following our theoretical model, in the epistemic continuum (Fig 1) sentence (2) *non so perché p* [I do not know why p] and sentence (3) *non so se p* [I do not know whether p] are contiguous, next to each other: unknowledge is adjacent to uncertainty. This theoretical, epistemic contiguity manifests itself at the linguistic level, being the two sentences much alike: they both include the negative verb expression *Non so* [I do not know]. The only difference between them is given by the adverb *perché* [why] and the conjunction *se* [whether].

Therefore, the theoretical, epistemic contiguity and linguistic likeness of the two sentences are not enough to explain why 50.75% of participants chose the unknowledge EMP for sentence (3). In our opinion, the decisive reason for this choice must be looked for in sentence (3) itself, i.e., in its epistemic complexity.

In the Theoretical framework section, sentence (3) *Non so se Andrea sta andando a Verona* [I do not know whether Andrea is going to Verona] was compared to the sentence *Non so se Andrea sta andando a Verona* ***o no*** [I do not know whether Andrea is going to Verona **or not**]: in both sentences the speaker’s uncertainty is between the alternative *p* (Andrea is going to Verona) and *non p* (Andrea is *not* going to Verona): which one is true? The only difference between the two sentences is that in *Non so se Andrea sta andando a Verona o no* [I do not know whether Andrea is going to Verona or not] the negative alternative *non p* is explicit, lexicalized as *o no* [or not], while in sentence (3) it remains implicit, not lexicalized. This implicitness could have led those participants (50.75%), who chose the unknowledge EMP (c) for sentence (3) *Non so se p* [I do not know whether p], to consider such a sentence as alike to sentence (2), i.e., as communicating unknowledge.

**Fig 9:**
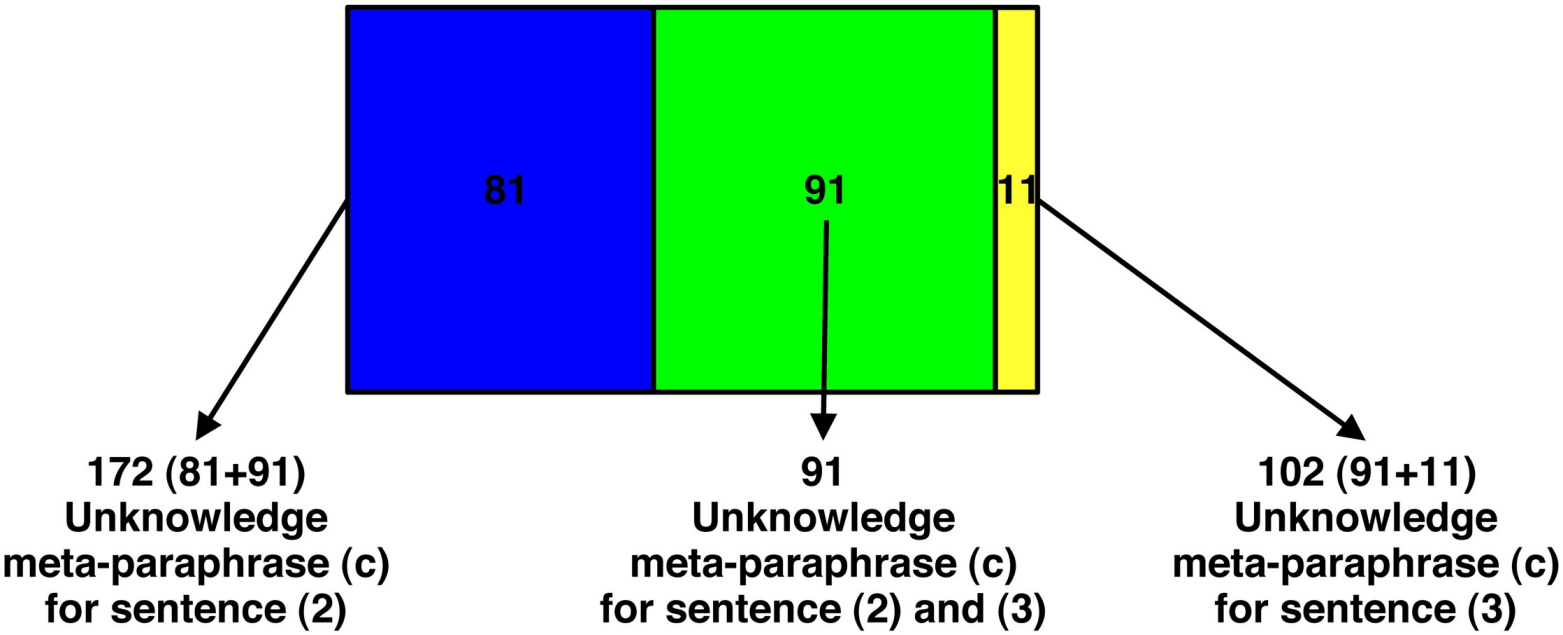
Participants who selected the unknowledge EMP for sentences (2) and (3). *The green section of the figure (i.e., the overlapping area) refers to subjects who assigned EMP (c) to both sentence (2) and sentence (3); the blue section of the figure refers to subjects who assigned EMP (c) to sentence (2), but not to sentence (3); the yellow section refers to subjects who assigned EMP (c) to sentence (3), but not to sentence (2).

This hypothesis seems to be supported by the number of the *same* participants (91) who chose the unknowledge EMP (c) for both sentence (2) and (3). As a matter of fact, the quantitative data (Table 2) show that 172 participants out of 201 (85.57%) chose the unknowledge EMP (c) for sentence (2) and that, as already we know, 102 participants out of 201 (50.75%) chose the same unknowledge EMP for sentence (3). These two sets of participants have in common 91 participants who chose the unknowledge EMP (c) for both sentence (2) and (3), as shown in Fig 9:

Fig 9 shows that 91 out 102 participants (89.22%), almost all participants who chose the unknowledge EMP (c) for sentence (3), chose the same EMP also for sentence (2); 91 out of 172 (52.91%) just over half of the participants who chose the unknowledge EMP (c) for sentence (2), chose the same EMP also for sentence (3). These 91 participants seem to give more weight to the negative verb expression *Non so* [I do not know] than to the adverb *perché* [why] or to the conjunction *se* [whether]. It is as if for them it makes no difference whether *Non so* [I do not know] is followed by *perché* [why] or by *se* [whether]. Their attention seems more focused on the negative verb expression than on what follows.

**Fig 10:**
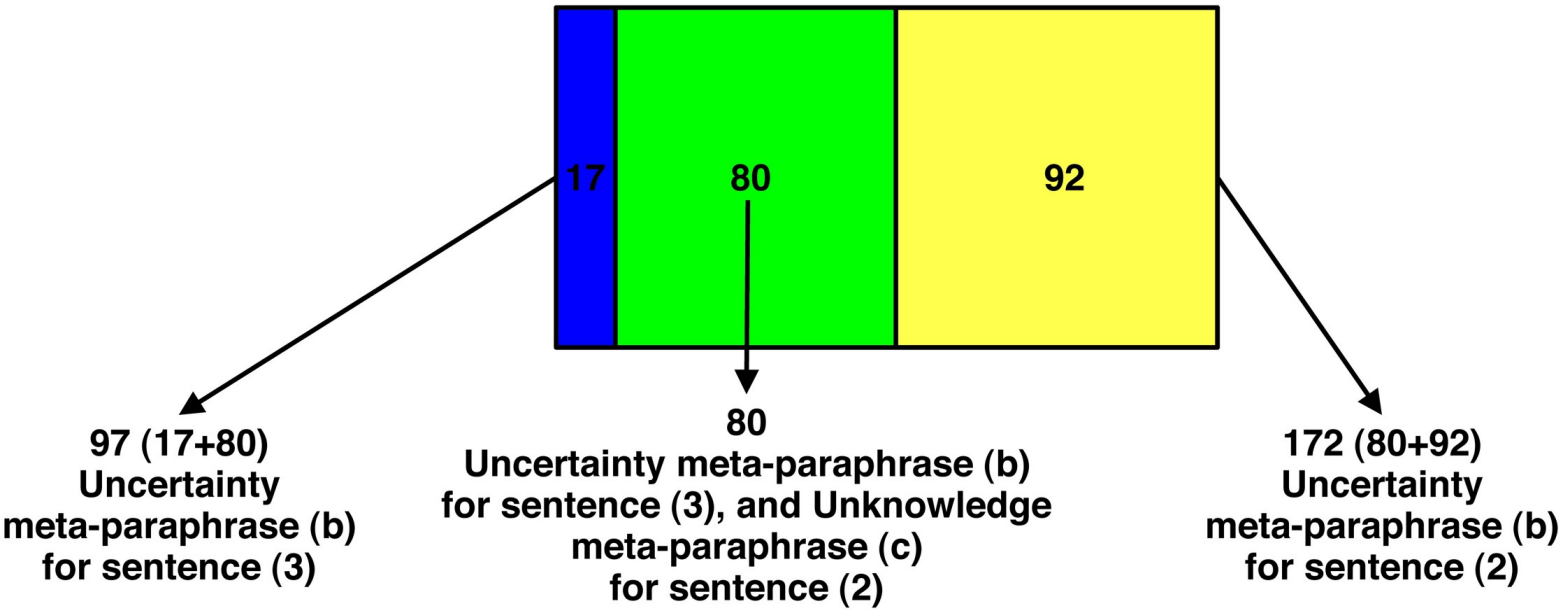
Participants who selected the uncertainty EMP for sentence (3) and the unknowledge EMP for sentences (2). *The green section of the figure (i.e., the overlap area) refers to the subjects who assigned the EMP (b) to sentence (3) and EMP (c) to sentence (2); the blue section of the figure refers to the subjects who assigned the EMP (b) to sentence (3); the yellow section refers to the subjects who assigned the EMP (b) to sentence (2).

Furthermore, if we go and see how many participants out of those 97 (48.25%, see Table 2), who chose the uncertainty EMP (b) for sentence (3) *Non so se p* [I do not know whether p], chose the unknowledge EMP (c) for sentence (2) *Non so perché p* [I do not know why p], we find also in this case a vast majority: 80 (82.47%). As a matter fact, the quantitative data (Table 3 and Fig 3) show that 97 participants out of 201 (48.25%) chose the uncertainty EMP for sentence (3) and that 172 out of 201 participants (85.57%) chose the unknowledge EMP (c) for sentence (2). These two sets of participants have in common 80 participants who chose the uncertainty EMP (b) for sentence (3) and the unknowledge EMP (c) for sentence (2), as shown in Fig 10:

Fig 10 shows that 80 out 97 (82.47%), i.e., almost all participants who chose the uncertainty EMP (b) for sentence (3), chose the unknowledge EMP (c) for sentence (2); 80 out of 172 (46.51%), i.e., slightly less than half of the participants who chose the unknowledge EMP (c) for sentence (2), chose the uncertainty EMP (b) for sentence (3). These 80 participants seem to give as much weight to the negative verb expression *Non so* [I do not know] as to *se* [whether] and *perché* [why]. They seem to distinguish between *not knowing whether* and *not knowing why*. It is as if for them it does make a difference if *Non so* [I do not know] is followed by *perché* [why] or by *se* [whethe*r*]. Their attention seems to focus on the negative verb expression as well as on what follows.

Finally, let us go back to 50.75% of participants who, unexpectedly for us, chose the unknowledge EMP (c) for sentence (3) *Non so se p* [I do not whether p]. We would like to emphasise also the data concerning those participants (24 out of 201 = 11.94%, Table 2, Fig 3) who, contrary to expectations, chose the *uncertainty* EMP (b) for sentence (2) *Non so perché p* [I do not know why p] as well as those participants who chose the *certainty* EMP (a) for sentence (4) *Mi sa che p* [To me it knows that p], sentence (5) *Credo che p* [I believe that p] and (6) *Penso che p* [I think that p] (specifically 23 out 201 = 11.44% for sentence (4) and 24 out of 201 = 11.94% respectively for sentences (5) and (6), Table 2, Fig 3).

Since we cannot simply ascribe this unexpected result to a low level of education, because the socio-demographic data show that the majority of participants (over 70%) have at least a BA (and almost 30% even a PhD or similar higher education qualification), another factor that could ideally be considered concerns the level of attention given by the participants to complete the task. Although this factor cannot be controlled and statistically analysed, considering the characteristics of the instrument used (the questionnaire did not require a specific time interval for completion, but the participants could interrupt and resume at any time), with all due caution and fully aware of the limitations of this data, we can however note that the average time taken to complete the questionnaire was 4.35 minutes (> 5 minutes for 73.6% of the participants). Thus it is possible to assume that a considerable part of the participants gave their answers in a rather immediate and intuitive manner, without leaving too much time for reflection. In this framework, the partial lexical overlap (***non so*** *perché*–***non so*** *se*) may have led some participants more likely to choose the unknowledge EMP for both sentence (2) and (3).

In conclusion, it could be interesting to investigate what reasoning led the participants to formulate their choice, but only an additional study, conducted with a different methodology, could give us satisfactory answers.

## Conclusions

The two studies presented in this paper concerning the Italian epistemic marker *mi sa* try to fulfil a gap in the literature on epistemic markers in the Italian language.

Study 1 answers the research question “how many are the occurrences of *mi sa* and of what types are they in the contemporary Italian spoken corpus KIParla [40]?”

The main findings of the qualitative and quantitative analysis refer to the identification of five types of occurrences, which can be reduced to two main ones, differing between them in meaning and morphology: *mi sa che* + proposition (the most numerous in KIParla, 97.9%, including the plain form *mi sa che* + proposition 60.6%, *mi sa* parenthetical 26.6%, *mi sa* + elliptical proposition 5.3%, *mi sa che* [pending] 4.3% and *mi sa di no* 1.1%) and *mi sa di* [metaphorical] (see Table 1 and Fig 2).

Study 1, although of theoretical interest in its own regard, as it was aimed both at extending Serianni’s study [38] and at exploring the possible uses of *mi sa* as an epistemic marker, also represents a corpus based, exploratory pilot study in respect of Study 2. As a matter of fact, the construction of the completive proposition used for the questionnaire in Study 2 was based on the most frequent grammatical configuration of the propositions introduced by *mi sa* in the corpus analysed.

The questionnaire presented in Study 2 was conceived to answer the following research questions:

1. which is the epistemic relationship between *mi sa* and the other modal expressions that use the verb *sapere* [to know] in the first person singular of the simple present, i.e., *so* [I know], *non so* [I do not know], *non so se* [I do not know whether]?
2. which are the epistemic relationships between *mi sa*, *credo* [I believe] and *penso* [I think]? Since they are supposed to be synonyms, what kind of evidence can be given to support such supposition?
3. within the specific epistemic continuum of uncertainty, which are the epistemic relationships between *non so se*, on the one side, and *mi sa*, *credo* and *penso*, on the other? Is the degree of uncertainty that they communicate the same or is it significantly different?

The quantitative and statistical results of the first part of the questionnaire (choosing an epistemic EMP for each of the six sentences) show the epistemic relationships between the six markers (research questions 1 and partially 2): as expected, for the majority of the participants

- sentence (1) *so che p* [I know that p] communicates the speaker’s *knowing/certain* position;
- sentence (2) *non so perché p* [I do not know why p] communicates the speaker’s *unknowing* position;
- sentences (4) *mi sa che p* [To me it knows that p], (5) *credo che p* [I believe that p], (6) *penso che p* [I think that p] come from the speaker’s *uncertain* position;
- sentence (3) *non so se p* [I do not know whether p] was expected to be referred to the uncertain position, but only for about half of the participants it comes from this position; for the second half, sentence (3) comes from the *unknowing* position. Some hypothetical explanations for these data are advanced in the Discussion.

The second part of the questionnaire (evaluating how much uncertainty sentences (3)-(6) communicate) answers research questions 3 (which evidence for the supposed synonymity of *mi sa*, *credo*, *penso*) and 4 (do these last three markers communicate the same degree of uncertainty as *non so se* or not?).

The quantitative and statistical results of the second part of the questionnaire give evidence for the supposed synonymity of *mi sa*, *credo*, *penso* and show that sentence (3) *Non so se p* is evaluated as highly uncertain, i.e., as much more uncertain than sentences (4) *Mi sa che p*, (5) *Credo che p*, (6) *Penso che p*, which are given about the same degrees of low uncertainty.

**Fig 11:**
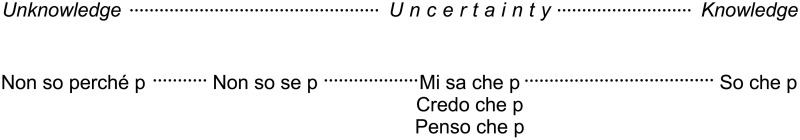
The six sentences along the epistemic continuum according to the results of Study 2.

Such results seem also to confirm that the epistemic continuum of uncertainty ranges between two poles, from high uncertainty (the *not knowing whether* pole, which sentence (3), read as a speaker’s doubt, refers to) to low uncertainty (the *believing* pole, which sentences (4)-(6), read as a speaker’s opinion, refer to). Our hypothesis concerning the placement of each epistemic marker on the epistemic stance continuum was confirmed (at least partially) by the results of our data analysis, in particular, by the participants’ choices of EMPs and by the degrees of uncertainty they assigned to the sentences for which they had chosen EMP (b), i.e., ‘the speaker communicates to be uncertain’. The only exception is represented by the sentence (3) *non so se* [I don’t know whether], which seems to be placed between unknowing and uncertainty, as the participants split in half in their evaluation. This leads us to partially modify the placement of the markers along the continuum, moving *non so se* to the left, in an intermediate space between Not Knowing and Uncertainty (Fig 11).

In the Discussion, the implicitness of the negative alternative *non p* (Andrea is *not* going to Verona) in sentence (3) *Non so se Andrea sta andando a Verona* [I do not know whether Andrea is going to Verona] was supposed to be, together with the epistemic contiguity and the linguistic likeness of sentences (2) and (3), the main reason why about 48% of participants chose the uncertainty EMP (b) for sentence (3). Thus, in a future study, instead of sentence (3), we intend to use the sentence *Non so se Andrea sta andando a Verona* ***o no*** [I do not know whether Andrea is going to Verona **or not**], where the negative alternative is explicit, in order to compare such sentence with sentence (2) *I do not know why* Andrea is going to Verona and to see whether this variable can favour the choice of the uncertainty EMP (b) for such new sentence. We did not do that in the present Study 2 since we wanted to keep *constant* the propositional content *Andrea is going to Verona*.

Among the limitations of Study 2 we can mention the fact that the questionnaire did not include any questions related to the geographical area(s) from which participants were sampled. This data could certainly have been very interesting to investigate, but it required a series of questions that in our opinion would have made the questionnaire much more complex. As a matter of fact, it is certainly not enough to know a subject’s geographical area of residence to be able to assess the possible impact of this variable on their choice, but other factors could be considered (e.g. the area in which one was born and/or grew up, if not coinciding to one’s area of residence; the area from which one’s parents come, etc.), also bearing in mind the wide differences in dialects found throughout Italy, where different dialects do not only concern different regions, but also provinces or even municipalities. A further study could be devoted to this topic.

Nevertheless, in spite of its limitations, the study certainly adds some insights to our understanding of the communication of uncertainty in the Italian language and contributes to investigating, with a multimethod approach, features and functions of an epistemic marker poorly studied in the literature.

## Supporting information

S1 Data (XLSX)

S1 Questionnaire (PDF)
