# Supplementary material for: The Italian epistemic marker mi sa [to me it knows] compared to so [I know], non so [I don’t know], non so se [I don’t know whether], credo [I believe], penso [I think]
Source: PLoS One. 2022 Sep 22;17(9):e0274694. doi: 10.1371/journal.pone.0274694 (PMC9499615; doi:10.1371/journal.pone.0274694)
Supplement: S1 Questionnaire — (PDF) [file pone.0274694.s002.pdf]

THE COMMUNICATION OF WHAT WE KNOW, WE DO NOT KNOW OR WE ARE UNCERTAIN ABOUT

We ask for your willingness to answer a short questionnaire on communication, the completion of which will take about 10 minutes. Your intuitive, natural competence as a speaker of the Italian language will be enough to answer the questions.
The study is conducted by a team of psychologists from the Universities of Macerata and Verona.

If you decide to accept, you will be asked to give your consent to the participation and processing of the data collected through the answers you wish to provide. The data will be processed in accordance with Article 13 of Regulation (EU) No. 679/2016 and Decree Law 196/2003, as adapted to Decree Law 101/2018 on the protection of personal data. The data will be collected anonymously and used exclusively for scientific purposes. All information collected will be stored securely and prevented from being viewed by unauthorised parties. The material will be kept by the persons responsible for the study. The results of the research will be made public both through the usual scientific channels (participation in national and international conferences and seminars, scientific publications) and through more popular channels.

We thank you in advance for your cooperation.

A1. **We ask for your willingness to answer a short questionnaire on communication, the completion of which takes about 10 minutes. It will consist of assessing 6 short sentences that will be presented to you in succession: your intuitive, natural competence as a speaker of the Italian language will be enough to answer the questions. The study is conducted by a team of psychologists from the Universities of Macerata and Verona. If you decide to accept, you will be asked to give your consent to the participation and processing of the data collected through the answers you provide. The data will be processed in accordance with Article 13 of Regulation (EU) no. 679/2016 and by Legislative Decree 196/2003, as adapted to Legislative Decree 101/2018 on the protection of personal data. The data will be collected anonymously and used exclusively for scientific research purposes.**
All information collected will be stored securely and prevented from being viewed by unauthorised parties. The material will be kept by the persons responsible for the study. The results of the research will be made public both through the usual scientific channels (participation in national and international conferences and seminars, scientific publications) and through more popular channels.

We thank you in advance for your cooperation.

I consent to participation and data processing.

Yes ☐

No ☐

B1. Gender

Male ☐

Female ☐

I prefer not to answer ☐

Other ☐

Other

☐

B2. Age

☐☐☐☐☐☐☐☐☐☐

B3. Level of education

Primary or middle school diploma ☐

High school diploma ☐

BA ☐

MA ☐

Postgraduate studies (PhD, specialisations, etc.) ☐

B4. Mother tongue

Italian ☐

Not Italian ☐

Bilingual (two mother tongues, one of which is Italian) ☐

C1. I know that Andrea is going to Verona

The speaker communicates to be certain that Andrea is going to Verona ☐

The speaker communicates not to be certain that Andrea is going to Verona ☐

The speaker communicates not to know that Andrea is going to Verona ☐

C2. Now evaluate how much uncertainty the sentence 'I know that Andrea is going to Verona' communicates, by using the scale ranging from 1 (very little uncertainty) to 10 (very much uncertainty).

There are no right or wrong answers, because nobody knows how things really are; that is why we are interested in knowing your point of view.

|1- Very little uncertainty |10- Very much uncertainty

D1. I do not know why Andrea is going to Verona

The speaker communicates to be certain about the reasons why Andrea is going to Verona ☐

The speaker communicates not to be certain about the reasons why Andrea is going to Verona ☐

The speaker communicates not to know the reasons why Andrea is going to Verona ☐

D2. **Now evaluate how much uncertainty the sentence 'I do not know why Andrea is going to Verona' communicates, by using the scale ranging from 1 (very little uncertainty) to 10 (very much uncertainty).**

There are no right or wrong answers, because nobody knows how things really are; that is why we are interested in knowing your point of view.

|1- Very little uncertainty |10- Very much uncertainty ☐☐☐☐☐☐☐☐☐☐

E1. I do not know whether Andrea is going to Verona

The speaker communicates to be certain that Andrea is going to Verona ☐

The speaker communicates not to be certain that Andrea is going to Verona ☐

The speaker communicates not to know that Andrea is going to Verona ☐

E2. **Now evaluate how much uncertainty the sentence 'I do not know whether Andrea is going to Verona' communicates, by using the scale ranging from 1 (very little uncertainty) to 10 (very much uncertainty).**

There are no right or wrong answers, because nobody knows how things really are; that is why we are interested in knowing your point of view.

|1- Very little uncertainty |10- Very much uncertainty ☐☐☐☐☐☐☐☐☐☐

F1. To me it knows that Andrea is going to Verona

The speaker communicates to be certain that Andrea is going to Verona ☐

The speaker communicates not to be certain that Andrea is going to Verona ☐

The speaker communicates not to know that Andrea is going to Verona ☐

F2. **Now evaluate how much uncertainty the sentence 'To me it knows that Andrea is going to Verona' communicates, by using the scale ranging from 1 (very little uncertainty) to 10 (very much uncertainty).**

There are no right or wrong answers, because nobody knows how things really are; that is why we are interested in knowing your point of view.

|1- Very little uncertainty |10- Very much uncertainty ☐☐☐☐☐☐☐☐☐☐

G1. **I believe that Andrea is going to Verona**

The speaker communicates to be certain that Andrea is going to Verona ☐

The speaker communicates not to be certain that Andrea is going to Verona ☐

The speaker communicates not to know that Andrea is going to Verona ☐

G2. **Now evaluate how much uncertainty the sentence 'I believe that Andrea is going to Verona' communicates, by using the scale ranging from 1 (very little uncertainty) to 10 (very much uncertainty).**

There are no right or wrong answers, because nobody knows how things really are; that is why we are interested in knowing your point of view.

|1- Very little uncertainty |10- Very much uncertainty ☐☐☐☐☐☐☐☐☐☐

H1. **I think that Andrea is going to Verona**

The speaker communicates to be certain that Andrea is going to Verona ☐

The speaker communicates not to be certain that Andrea is going to Verona ☐

The speaker communicates not to know that Andrea is going to Verona ☐

H2. **Now evaluate how much uncertainty the sentence 'I think that Andrea is going to Verona' communicates, by using the scale ranging from 1 (very little uncertainty) to 10 (very much uncertainty).**

There are no right or wrong answers, because nobody knows how things really are; that is why we are interested in knowing your point of view.

|1- Very little uncertainty |10- Very much uncertainty

THANK YOU FOR PARTICIPATING
